# COVID-19 vaccine-induced autoimmune hyperthyroidism: Graves’ disease

**DOI:** 10.3389/fimmu.2025.1699210

**Published:** 2025-12-03

**Authors:** Avaniyapuram Kannan Murugan, Ali S. Alzahrani

**Affiliations:** 1 Division of Molecular Endocrinology, Department of Molecular Oncology, King Faisal Specialist Hospital and Research Centre, Riyadh, Saudi Arabia; 2 Department of Medicine, King Faisal Specialist Hospital and Research Centre, Riyadh, Saudi Arabia

**Keywords:** thyroid, COVID-19 vaccine, hyperthyroidism, autoimmune, SARS-CoV-2, Graves’ disease, inflammatory syndrome, ACE2

## Abstract

Graves’ disease (GD) is an autoimmune disorder that results in hyperthyroidism, in which the immune system mistakenly targets the thyroid gland, causing it to produce excessive amounts of thyroid hormones. Genetic predisposition, environmental factors such as infections and stress, disruptions in the gut microbiome, excessive iodine intake, and epigenetic changes have all been implicated in the development of GD. The recent pandemic caused by severe acute respiratory syndrome coronavirus 2 (SARS-CoV-2) posed a serious global health crisis. The emergence of COVID-19 vaccines has been pivotal in combating the viral infection and its spread. However, reports of rare adverse events, including the development of autoimmune disorders such as GD following vaccination, have raised concerns. Autoimmune factors play a critical role in the pathogenesis of GD, particularly through the production of autoantibodies targeting the thyroid gland. In this review, reported cases are critically analyzed to elucidate commonalities and potential triggers for the development of this autoimmune disorder, highlighting the vital role of autoimmune mechanisms in inducing GD. We also discuss the molecular mechanisms underlying vaccine-induced autoimmunity, including antigen presentation, bystander activation, molecular mimicry, and the induction of inflammatory factors following vaccination. Understanding these mechanisms in COVID-19 vaccine-induced GD could enhance patient care and guide vaccination policies.

## Introduction

Coronavirus disease 2019 (COVID-19) is a respiratory illness caused by severe acute respiratory syndrome coronavirus 2 (SARS-CoV-2), first identified in Wuhan, China, in December 2019. It rapidly escalated into a global outbreak, leading the World Health Organization (WHO) to declare it a pandemic in March 2020. The virus primarily transmits through respiratory droplets released when an infected individual coughs, sneezes, or speaks. Symptoms can range from mild, such as fever, fatigue, and cough, to more severe complications, including pneumonia and difficulty breathing (1). The SARS-CoV-2 “spike” (S) glycoprotein uses human angiotensin-converting enzyme 2 (ACE2) as a receptor for cellular entry, facilitating infection. ACE2 is highly expressed in various vital organs, including the heart, lungs, kidneys, blood vessels, small intestine, thyroid, and parathyroid glands (2, 3). Endocrine tissues, particularly the thyroid and parathyroid, are therefore likely to be affected by SARS-CoV-2. Viruses like SARS-CoV-2 preferentially infect cells with high ACE2 expression, such as thyroid and parathyroid cells, enabling rapid viral replication in these tissues. The immune system, in attempting to eliminate the virus, may inadvertently attack ACE2-expressing cells. This misdirected response can potentially lead to subsequent autoimmune complications. Consequently, the SARS-CoV-2 infection has been shown to induce various thyroid pathogeneses, including subacute thyroiditis, Graves’ disease (GD), thyroid sick syndrome, and Hashimoto’s thyroiditis (4, 5). Recently, a similar effect was also observed in the parathyroid gland (3). Overall, older adults, individuals with obesity, and those with preexisting chronic health conditions are at higher risk of severe illness.

As of 9 February 2025, a total of 777,385,370 COVID-19 cases had been reported globally, resulting in 7,088,757 deaths (1).

Multiple strategies, including vaccines, public health measures, and treatments, have been developed to combat the spread of SARS-CoV-2 and mitigate its impact. The rapid development and deployment of COVID-19 vaccines have been pivotal in reducing the pandemic’s severity (6). Moreover, these vaccines have significantly decreased illness severity and mortality caused by SARS-CoV-2 (7). Vaccinated individuals also have a lower risk of developing postacute sequelae of COVID-19 (PASC), commonly known as long COVID, highlighting another key benefit of vaccination (8). Since the introduction of COVID-19 vaccines, a total of 13.64 billion doses have been administered worldwide. By 31 December 2023, 67% of the global population had completed the full primary series of a COVID-19 vaccination. Additionally, 32% of the world’s population had received at least one booster dose (9). The rapid development and deployment of vaccines targeting SARS-CoV-2, the virus responsible for COVID-19, have played a crucial role in curbing the spread of the pandemic. However, COVID-19 vaccines have been associated with rare acute adverse events, including myocarditis, pericarditis, thrombosis, thrombocytopenia, Guillain–Barré syndrome, transverse myelitis, and Bell’s palsy (10). One such potential complication is the development of autoimmune thyroid disorders, including GD. According to the WHO, GD is an autoimmune disorder characterized by the overproduction of thyroid hormones (thyrotoxicosis) due to circulating autoantibodies that stimulate the thyroid-stimulating hormone receptor or thyrotropin receptor (TSHR). Symptoms can include weight loss, rapid heartbeat, anxiety, tremors, heat intolerance, diffuse goiter, bulging eyes (Graves’ orbitopathy), and, less commonly, dermopathy. Suppressed thyroid-stimulating hormone (TSH) levels, along with elevated free T4 and/or T3 and positivity for TSHR autoantibodies (TRAb), are indicative of GD. Imaging findings typically show diffusely increased radioactive iodine uptake (RAIU) and enhanced blood flow on thyroid ultrasonography with color Doppler, often described as a “thyroid inferno”. Treatment options include antithyroid medications, radioactive iodine therapy, or surgical intervention (9, 11).

This review aims to summarize the current evidence on the emergence of GD following SARS-CoV-2 vaccination, with a focus on the underlying autoimmune and inflammatory processes. By elucidating the pathophysiology and clinical implications of this phenomenon, it may help improve vaccination strategies and patient management during the ongoing COVID-19 pandemic.

## Postvaccine syndrome

Some individuals have reported experiencing postvaccination symptoms similar to long-COVID shortly after receiving the COVID-19 vaccine. This condition, known as postvaccination syndrome (PVS) or postacute COVID-19 vaccination syndrome (PACVS), is characterized by symptoms such as extreme fatigue, reduced exercise tolerance, numbness, brain fog, nerve pain, insomnia, palpitations, muscle pain, tinnitus, headaches, burning sensations, and dizziness (12, 13). Unlike long-COVID, PVS is not officially recognized by health authorities, which limits access to appropriate patient care and support. The underlying molecular mechanisms of PVS remain largely unclear.

However, there is a significant overlap in self-reported symptoms between long-COVID and PVS, both of which involve exposure to the SARS-CoV-2 “S” protein in the context of immune responses triggered by infection or vaccination (13, 14). In individuals with underlying susceptibility, vaccines might trigger persistent symptoms through various biological mechanisms. First, vaccine components such as mRNA, lipid nanoparticles, and adenoviral vectors activate pattern recognition receptors, potentially leading to excessive immune activation and chronic inflammation (15, 16). Additionally, studies have shown that the “S” protein produced after BNT162b2 or mRNA-1273 vaccination can circulate in plasma within a day of vaccination (17). Interactions between the full-length “S” protein, its subunits (S1, S2), and peptide fragments with host molecules may contribute to prolonged symptoms in certain individuals (17). Recent research has also identified a subset of nonclassical monocytes containing the “S” protein in patients with PVS (18). Furthermore, animal studies on mRNA–lipid nanoparticle (LNP) platforms suggest that these components can cross the blood–brain barrier, leading to localized “S” protein expression that may contribute to neurocognitive symptoms (19). Lastly, vaccine-induced immune responses may stimulate autoreactive lymphocytes, potentially triggering autoimmune reactions (20). Recent studies and case reports have raised concerns about a possible link between COVID-19 vaccination and the development of GD (11, 21). While the exact mechanisms remain unclear, it is hypothesized that the immune response triggered by the vaccine may lead to the production of autoantibodies targeting the thyroid gland, resulting in hyperthyroidism and the clinical manifestations of GD. Moreover, many other factors are implicated in the development of classical GD. Understanding the potential association between COVID-19 vaccination and autoimmune thyroid disorders is essential for managing vaccinated individuals who develop GD.

## Factors implicated in inducing Graves’ disease

Graves’ disease is a complex autoimmune disorder influenced by multiple factors. Although its exact cause remains unknown, several factors have been implicated in triggering the development of GD.

### Genetic factors

Genetic predisposition plays a significant role in the development of GD. Certain genetic variations are associated with an increased risk of the condition, with genetic factors contributing to ~ 70%–80% of autoimmune thyroid disease (AITD). Variants in certain genes involved in immune regulation have been implicated in GD. For instance, human leukocyte antigen (*HLA*) genes encode proteins that present antigens to T cells, which are crucial for immune recognition. Specific HLA class II alleles (e.g., HLA-DR3) are strongly associated with GD, as they may present self-antigens, like TSHR peptides, more effectively to immune cells, thereby promoting an autoimmune response. Cytotoxic T-lymphocyte-associated protein 4 (*CTLA4*) is a checkpoint inhibitor that downregulates T-cell activity and helps maintain self-tolerance. Variants in the *CTLA4* gene may impair this inhibitory function, allowing autoreactive T cells to proliferate and attack the thyroid. Protein tyrosine phosphatase nonreceptor type 22 (*PTPN22*) genes regulate T-cell activation by negatively modulating signaling pathways. The R620W polymorphism in PTPN22 is associated with several autoimmune diseases and likely disrupts T-cell regulation, enhancing autoimmunity and contributing to the development of GD (22). In addition, non-HLA genes have also been associated with GD. For example, the B-cell surface antigen CD40 (*CD40*) is involved in B-cell activation and antibody production under normal physiological conditions. Variants in CD40 have been shown to enhance B-cell activation and promote the production of TRAb, which stimulates the thyroid. The TSHR regulates normal thyroid hormone synthesis, and polymorphisms in the *TSHR* gene may lead to abnormal expression or structural changes, rendering it more immunogenic and triggering the production of autoantibodies against it. Moreover, thyroglobulin (TG) is a key protein in thyroid hormone synthesis and can serve as a potential autoantigen. Certain TG gene variants may increase the likelihood of it being recognized as foreign, triggering an autoimmune attack (23). These genetic variants could disrupt immune tolerance and promote the development of autoantibodies, particularly against TSHR, which is central to GD pathogenesis. The combination of immune dysregulation and enhanced presentation of thyroid autoantigens underlies the autoimmune nature of GD.

### Epigenetic factors: methylation, miRNAs, and lncRNAs

Epigenetic mechanisms, which involve changes in gene expression without alterations to the underlying DNA sequence, have been implicated in the development of GD. These changes can be influenced by environmental factors and may contribute to the dysregulation of immune responses observed in GD (24). Various epigenetic mechanisms, including DNA methylation—such as rs2228612 in DNA-methyltransferase 1 (DNMT1)—are associated with DNA hypomethylation and GD intractability (25). Regarding histone modifications, several genes, including *CD247*, *CD3D*, *CD3E*, *CD3G*, *CD8A*, *LCK*, *ZAP70*, and *CTLA4*, show decreased H3K4me3 levels in their promoters, leading to downregulation of their expression in both CD4+ and CD8+ T cells of GD cases (26). Many noncoding RNAs, including microRNAs (miRNAs) and long noncoding RNAs (lncRNAs), have also been implicated in the development of GD. For instance, altered expression of let-7b and miR-146a-5p has been associated with GD onset (27). Additionally, the long noncoding RNA Heg has been found to reduce CD14 mRNA levels in mononuclear cells of individuals with GD (28). However, the same group reported that antithyroid treatment did not affect lncRNA Heg levels in GD cases, suggesting that further studies are needed to clarify this relationship (29). Furthermore, exosomes have also been implicated in the development of GD. For instance, exosomes derived from thyrocytes, which target dendritic cells and contain TPO, heat shock protein 60, major histocompatibility complex (MHC) class II, and activated dendritic cells, can significantly enhance CD4+ T lymphocyte responses, contributing to the onset and progression of AITD (30).

### Stress and psychological factors

Stress and psychological factors have been implicated in the development of autoimmune diseases, including GD. Stress can impact immune function and may contribute to the development or exacerbation of autoimmune responses. Studies have shown that people with posttraumatic stress disorder (PTSD) are more likely to develop GD, highlighting the need for further research into the relationship between PTSD and thyroid disorders (31). Stress has also been associated with various other autoimmune conditions, including GD (32). The proposed mechanisms include activation of the hypothalamic–pituitary–adrenal (HPA) axis and the sympathetic nervous system, leading to altered cytokine profiles and immune dysregulation. Chronic stress may also impair regulatory T-cell function, thereby increasing the risk of autoantibody production against thyroid antigens.

### Gut microbiome

Disruption of the gut microbiome, known as dysbiosis, may contribute to immune dysregulation and autoimmune diseases such as GD (33, 34). A relationship between GD and *Yersinia enterocolitica* has been reported; mice exclusively fed with *Y. enterocolitica* did not develop GD (35). Although some studies have examined gut microbiota in hyperthyroid patients, research specifically linking GD to gut microbiota remains limited (36). Thyroid hormone levels have also been shown to influence gut microbiota composition in GD patients (37). Bacteroidetes and Firmicutes dominate the gut microbiome. In GD, the proportion of Bacteroidetes increases while Firmicutes decrease, resulting in reduced alpha diversity (38–41). Other microbial shifts associated with GD include increases in Lactobacillales, Bacilli, Megamonas, Prevotella, and Veillonella, and decreases in Rikenellaceae, Ruminococcus, and Alistipes (42). Moreover, patients with GD show lower overall microbial richness (42). Bacteroides, Prevotella, and Alistipes have been shown to distinguish GD patients from healthy controls with 85% accuracy (43). TRAb, a key marker of GD, exhibits over 95% sensitivity and specificity (44). TRAb levels positively correlate with *Succinivibrionaceae* and *Subdoligranulum* and negatively with *Parabacteroides distasonis* (45). TRAb-positive patients may also have a higher risk of Graves’ orbitopathy (GO) (45). Further studies are needed to confirm interactions between the thyroid and gut microbiome.

### Iodine intake

Excessive iodine intake has long been associated with the development of autoimmune thyroid disorders, including GD. While high iodine intake usually leads to mild and short-term thyroid issues, it can trigger severe and potentially life-threatening hyperthyroidism in some individuals (46). Iodine is essential for thyroid hormone production, and excessive iodine intake can trigger or exacerbate thyroid autoimmunity in susceptible individuals (47). The proposed mechanism may involve, firstly, increased iodination of thyroglobulin, which could enhance its immunogenicity and trigger an autoimmune response. Secondly, excess iodine can induce oxidative stress and apoptosis in thyroid cells, resulting in the release of autoantigens that activate autoreactive T and B cells.

### Dietary factor: selenium

Certain dietary factors, particularly selenium deficiency or excess, have been suggested to influence the development of GD. Selenium is a vital trace element essential for maintaining proper thyroid function and supporting immune system regulation. Several studies have shown that selenium deficiency is a risk factor for the development of GD (48). Mechanistically, selenium deficiency impairs the activity of glutathione peroxidase and other selenoproteins, resulting in increased oxidative stress in thyroid tissue. This oxidative damage can promote the release of autoantigens and disrupt immune tolerance, contributing to the initiation of autoimmune responses against the thyroid. Conversely, excess dietary selenium can result in selenosis, characterized by symptoms such as hair loss, brittle nails, nausea, and fatigue. In severe cases, it may cause neurological damage and gastrointestinal disturbances (33).

### Vitamin D deficiency

Vitamin D deficiency has been associated with an increased risk of autoimmune diseases, including GD. Vitamin D plays an important role in immune function and may help regulate the immune response in AITD (49, 50). A high prevalence of GD has been observed in vitamin D-deficient women (50), and low serum vitamin D levels have been reported in GD cases with no remission (51), collectively indicating that vitamin D has a considerable impact on GD. While these factors have been implicated in the development of GD, it is important to note that the underlying mechanisms of the disease are complex and multifactorial. Vitamin D deficiency can impair regulatory T-cell function and promote the activation of proinflammatory T helper (Th)1 and Th17 cells, resulting in an enhanced autoimmune response. Additionally, low vitamin D levels may increase the expression of MHC class II molecules and costimulatory signals on antigen-presenting cells, thereby enhancing autoantigen presentation and thyroid-specific immune activation (50).

### Viruses

Several viruses have been linked to an increased risk of autoimmune thyroid disorders, including GD. For instance, Epstein–Barr virus (EBV) infects B cells and triggers autoimmunity. Hepatitis C virus (HCV) has been associated with thyroid dysfunction in infected individuals (3, 4). Coxsackievirus has been implicated in autoimmune thyroiditis and possibly GD, while human herpesvirus-6 (HHV-6) has also been reported in some autoimmune disorders. Recent evidence suggests that SARS-CoV-2 infections may trigger thyroid autoimmunity, including GD (4).

### Vaccines

Several vaccines have been reported to potentially induce or exacerbate GD. Particularly, influenza vaccines, for example, activate the immune system and may trigger autoimmune thyroid disorders, particularly in individuals with a genetic or immunological predisposition. The hepatitis B vaccine, through immune responses to its recombinant proteins or adjuvants, may stimulate the production of thyroid autoantibodies (anti-TPO, anti-TG, or TRAb) (52). Similarly, the human papillomavirus (HPV) vaccine can activate the immune system via adjuvants (e.g., aluminum). Immune activation following yellow fever vaccination may also lead to the production of thyroid-stimulating immunoglobulins (TSI) (4).

## Chemical constituents and characteristics of various SARS-CoV-2 vaccines

**Figure 1:**
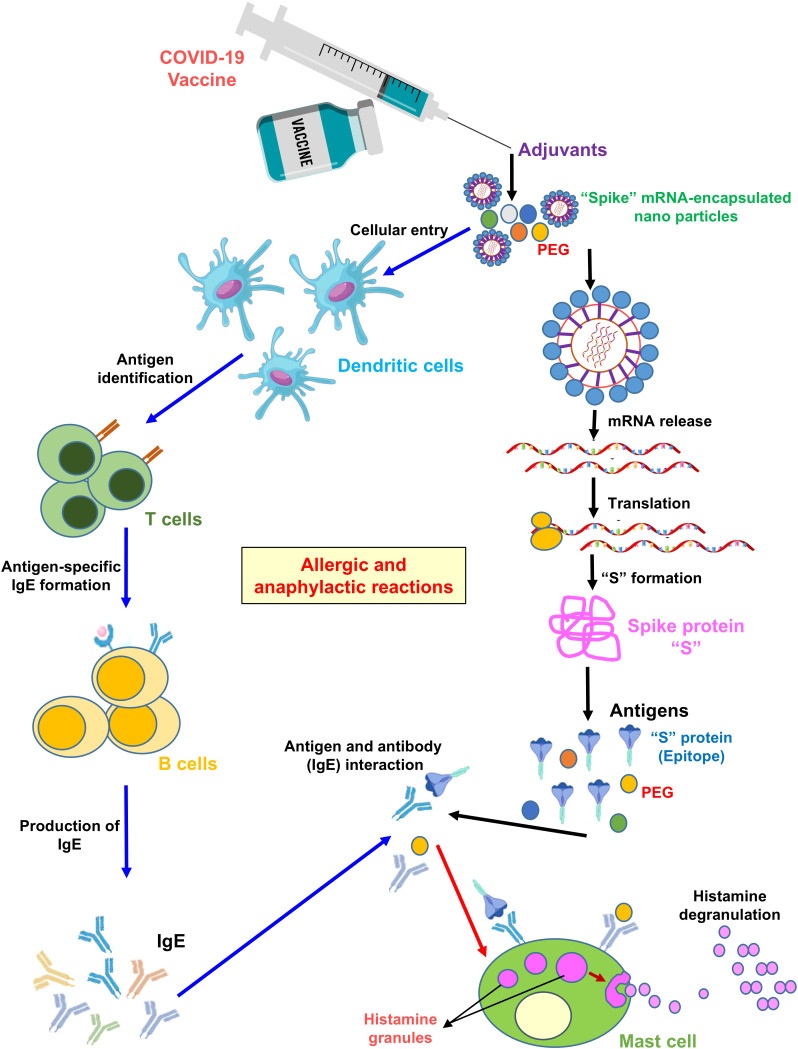
Mechanism of allergic and anaphylactic reactions following mRNA vaccination. As shown in the illustration, PEG nanoparticle packaging of the “S” protein mRNA enters an APC cell, such as dendritic cells, via endocytosis. The mRNA is then translated into the S protein by ribosomes. The APC presents free-floating PEG or S protein epitopes as antigens to T helper cells. The latter secrete cytokines, leading to B-cell activation. B cells produce IgE antibodies against PEG or S protein epitopes. Antigen-specific (PEG and S protein specific) IgE antibodies bind to the FcϵRI receptor. Engagement of this receptor triggers histamine release from basophils and/or mast cells, resulting in allergic/anaphylactic reactions.

As listed in **Table 1**, a total of 242 COVID-19 vaccines have been developed to date. Among these, 66 underwent phase I trials, 72 proceeded to phase II, and 92 reached phase III trials, while 12 vaccines were discontinued during the process. Of the 242 vaccines, 49 are currently in widespread use for COVID-19 immunization (11 approved by the WHO and 38 approved only in a few countries), and one was withdrawn after initial approval. Each vaccine contains unique active ingredients, excipients, and, in some cases, adjuvants, which may influence their effects in immunized individuals. There are three major types of vaccines administered against COVID-19: mRNA-based vaccines, inactivated virus vaccines, and viral vector-based vaccines. The mRNA-based vaccines include Pfizer-BioNTech^®^ BNT162b and Moderna^®^ mRNA-1273. These vaccines use nucleoside-modified mRNA encoding the full-length SARS-CoV-2 “S” protein, encapsulated in lipid nanoparticles. Notably, no adjuvants are included, as the mRNA itself elicits a strong immune response. Excipients consist of polyethylene glycol (PEG)-based lipids, sucrose for stabilization, and buffers to maintain pH. As illustrated in **Figure 1**, these vaccines may trigger allergic or anaphylactic reactions, primarily due to PEG components, which are known to cause hypersensitivity in susceptible individuals. Inactivated-virus vaccines include the Sinovac COVID-19 vaccine (Vero cell) and Covaxin. These vaccines contain a chemically inactivated SARS-CoV-2 virus as the active component, inducing immunity without viral replication. Aluminum hydroxide (Alum) is used as an adjuvant to enhance immunogenicity, while excipients such as phosphate buffers and sodium chloride help maintain isotonicity. The presence of adjuvants in vaccines is primarily associated with local injection-site reactions and systemic responses such as fever and fatigue. Viral vector-based vaccines include ChAdOx1 nCoV-19 (AstraZeneca) and Jcovden™ (Janssen). These vaccines use genetically modified adenoviruses—chimpanzee adenovirus for AstraZeneca and human adenovirus type 26 for Janssen—to deliver the SARS-CoV-2 “S” protein. They do not contain adjuvants, as viral vectors inherently stimulate innate immune pathways. Excipients include polysorbate 80, histidine, and various salts to stabilize the formulation. These vaccines can produce various effects, including injection-site irritation, systemic immune responses, and, in rare cases, hypersensitivity reactions. mRNA vaccines use lipid nanoparticle delivery, which triggers immune activation with minimal excipients but carries a risk of PEG-induced allergies. Inactivated-virus vaccines require adjuvants, such as aluminum hydroxide, to enhance the immune response, often causing localized inflammation. Viral vector vaccines utilize adenoviral delivery systems to induce immunogenicity, with excipients ensuring stability, though they pose a risk of systemic inflammatory reactions (53). **Table 1** summarizes differences in vaccine design, immunogenic components, and potential adverse effects, providing a clearer understanding of their safety profiles. To identify literature on COVID-19 vaccine-induced GD, we used the following search strategies.

**Table 1:** Constituents and characteristics of the COVID-19 vaccines.

S. No.	Vaccine name	Trade name	Manufacturer	Active ingredient	Adjuvant	Excipients	Possible side effects	WHO approval status
1	BNT162b2	Comirnaty	Pfizer-BioNTech^®^ (New York, USA)	A 5′-capped mRNA encoding the full-length SARS-CoV-2 spike protein	None	ALC-0315 (4-hydroxybutyl)azanediyl)*bis*(hexane-6,1-diyl)bis(2-hexyldecanoate), ALC-0159 (2-[(polyethylene glycol)-2000]-*N*,*N*-ditetradecylacetamide), 1,2-distearoyl-sn-glycero-3-phosphocholine (DSPC), cholesterol, potassium chloride, potassium dihydrogen phosphate, sodium chloride, disodium phosphate dihydrate, sucrose, and water.	Injection-site pain, fatigue, headache	WHO approved
2	mRNA-1273	Spikevax, mNexspike	Moderna^®^ (Cambridge, USA)	Nucleoside-modified mRNA encoding the viral spike (S) glycoprotein of SARS-CoV-2.	None	PEG2000-DMG: 1,2-dimyristoyl-rac-glycerol, methoxypolyethylene glycol, 1,2-distearoyl-sn-glycero-3-phosphocholine BotaniChol^®^ (nonanimal origin cholesterol) SM-102: heptadecane-9-yl 8-((2-hydroxyethyl) (6-oxo-6-(undecyloxy) hexyl) amino) octanoate, sodium acetate, sucrose, tromethamine, tromethamine hydrochloride, acetic acid.	Similar to BNT162b2	WHO approved
3	AZD1222	Vaxzevria, Covishield	AstraZeneca/Oxford (Cambridge, UK)	Recombinant, replication-deficient chimpanzee adenovirus vector encoding the SARS-CoV-2 spike glycoprotein, produced in genetically modified human embryonic kidney (HEK) 293 cells.	None	l-Histidine, l-histidine hydrochloride monohydrate, magnesium chloride hexahydrate, polysorbate 80 (E 433), ethanol, sucrose, sodium chloride, disodium edetate dehydrate, and water.	Flu-like symptoms, rare thrombotic events	WHO approved
4	Ad26.COV2.S	Janssen, Jcovden, JNJ-78436735, Ad26COVS1, VAC31518, Johnson & Johnson COVID−19 vaccine	Johnson & Johnson (New Brunswick, USA)	Nonreplicating viral vector. Adenovirus type 26 encoding the SARS-CoV-2 spike glycoprotein (Ad26.COV2-S).	None	Contains 2-hydroxypropyl-β-cyclodextrin (HBCD), citric acid monohydrate, ethanol, hydrochloric acid (for pH adjustment), polysorbate 80, sodium chloride, sodium hydroxide (for pH adjustment), trisodium citrate dihydrate, water for injection.	Similar plus rare thrombosis	WHO approved
5	BBIBP-CorV	Sinopharm BIBP COVID-19 vaccine, BIBP vaccine, Sinopharm COVID-19 vaccine	Sinopharm (Beijing, China)	Inactivated virus	Aluminum salts	Standard buffers	Local pain, mild fever	WHO approved
6	CoronaVac	Sinovac COVID-19 vaccine	Sinovac (Beijing, China)	Inactivated SARS-CoV-2 virus (CZ02 strain)	Aluminum salts	Disodium hydrogen phosphate dodecahydrate, sodium dihydrogen phosphate monohydrate, sodium chloride.	Mild local/systemic reactions	WHO approved
7	BBV152	Covaxin^®^	Bharat Biotech, in collaboration with the Indian Council of Medical Research—National Institute of Virology (Hyderabad, India)	Whole-virion inactivated. COVAXIN^®^ was developed using an inactivated/killed virus, along with the above-mentioned chemicals.	Aluminum salts	Contains 6 µg of whole-virion inactivated SARS-CoV-2 antigen (strain: NIV-2020-770), and other inactive ingredients, including aluminum hydroxide gel (250 µg), TLR 7/8 agonist (imidazoquinolinone, 15 µg), 2-phenoxyethanol (2.5 mg), and phosphate-buffered saline up to 0.5 mL	Injection-site pain, fever	WHO approved
8	NVX-CoV2373/TAK-019	Nuvaxovid, Covovax, Novavax COVID-19 vaccine	Novavax/Serum Institute of India (Pune, India)	Recombinant subunit	Matrix-M	Standard buffers	Tenderness, fatigue	WHO approved
9	Ad5-nCoV	Convidecia	CanSino Biologics (Tianjin, China)	Viral vector	None	Standard buffers	Injection-site pain, mild fever	WHO approved
10	Corbevax	BECOV2D, Biological E COVID-19	Biological E Ltd. (Hyderabad, India)	Recombinant subunit	CpG1018 +Alum	Standard buffers	Mild local/systemic reactions	WHO approved
11	VLA2001	Valneva COVID-19 vaccine	Valneva SE, in collaboration with the American biopharmaceutical company Dynavax Technologies. (Emeryville, USA)	Inactivated virus	Alum + CpG1018	Standard buffers	Injection-site pain, fatigue	WHO approved
12	CIGB-66	Abdala	CIGB (Cuba) (Havana, Cuba)	Protein subunit	Unknown	Standard buffers	Local reactions, fatigue	National approval^a^
13	ZF2001	Zifivax	Anhui Zhifei (Beijing, China)	Protein subunit	Alum	Standard buffers	Mild local/systemic symptoms	National approval
14	GBP510	Skycovione	SK Bioscience (Gyeonggi-do, South Korea)	Protein subunit	AS03	Standard buffers	Injection-site pain, headache	National approval
15	SCB-2019	Clover	Clover Biopharma (Sichuan, China)	Protein subunit	CpG1018 + Alum	Standard buffers	Fever, fatigue	National approval
16	Kostaive	LUNAR−COV19, Zapomeran	Arcturus Therapeutics (San Diego, USA)	Self-amplifying RNA vaccine	No adjuvant, lipid nanoparticles (LUNAR^®^-style)	Lipid nanoparticles and buffer components	Injection-site pain/tenderness, fatigue, headache, muscle/joint pain, chills, dizziness, fever	National approval
17	Convidecia	PakVac, Convidecia Air	CanSino Biologics Inc. (Tianjin, China)	Adenoviral vector, Ad5−nCoV	None	Mannitol, sucrose, polysorbate 80, sodium chloride, magnesium chloride, HEPES, glycerol, water for injection	Injection-site pain, fever, fatigue, headache, muscle/joint pain. The inhaled version shows fewer side effects	National approval
18	FINLAY-FR-2	SOBERANA 02, Pasteurcovac, Pasto Covac	Finlay Institute (Havana, Cuba)	Protein subunit	Unknown	Standard buffers	Local pain, mild fever	National approval
19	FINLAY-FR-1A	SOBERANA^®^ PLUS ST	Finlay Institute (Havana, Cuba)	Protein subunit	Unknown	Standard buffers	Booster-related effects	National approval
20	DB16439	Aurora-CoV	Vector Institute (Koltsovo, Russia)	Peptide vaccine	Unknown	Standard buffers	Low reactogenicity	National approval
21	Chumakov COVID-19	CoviVac^®^	Chumakov Center (Moscow, Russia)	Inactivated virus	Alum	Standard buffers	Mild local reactions	National approval
22	COVIran Barekat	Barakat	Shifa Pharmed (Tehran, Iran)	Inactivated virus	Alum	Standard buffers	Injection-site pain/tenderness, fever, headache, muscle/joint pain, chills	National approval
23	QazVac	QazCovid−in	Kazakhstan Research Institute for Biological Safety Problems (Zhambyl, Kazakhstan)	Inactivated virus (β−propiolactone-inactivated)	Alum	Standard buffers	Mild/moderate: injection-site pain/tenderness, headache, muscle pain, nausea, occasional fever	National approval
24	Noora	Noora Vaccine™	Bagheiatallah University of Medical Sciences (Tehran, Iran)	Truncated SARS−CoV−2 spike receptor-binding domain (RBD), nucleoprotein, and S1 fragments	Alum	Standard buffers	Local pain in ~ 80%, mild redness or swelling; no serious adverse events	National approval
25	ReCOV	Recombinant bicomponent COVID−19 vaccine	Jiangsu Rec-Biotechnology Co. Ltd. (Taizhou, China)	NTD−RBD−foldon (trimeric spike protein fragment) expressed in CHO cells	BFA03—a proprietary oil-in-water emulsion containing squalene and α−tocopherol	Oil-in-water emulsions (buffer, stabilizers, emulsifiers)	Injection pain, headache, fatigue	National approval
26	V-01	Likang^®^	Livzon Mabpharm Inc. (Zhuhai, China)	Protein subunit	IFN−PADRE−RBD−Fc dimer—an RBD-based antigen linked to interferon-α, PADRE epitope, and Fc fragment	Aluminum hydroxide (Alum)	Injection-site pain, headache, fatigue	National approval
27	MVC-COV1901	None	Medigen Vaccine Biologics Corporation (Taipei, Taiwan)	SARS-CoV-2 spike protein (S-2P) produced in CHO cells	CpG 1018 (TLR9 agonist) and aluminum hydroxide	Phosphate-buffered saline (PBS), polysorbate 80, sodium chloride, and other stabilizers	Mild to moderate: injection-site pain, fatigue, headache, muscle ache, fever (rare)	National approval
28	CHO cell, NVSI-06-08	None	National Vaccine and Serum Institute, a subsidiary of Sinopharm (Beijing, China)	Hybrid mutI-tri-RBD (fusion of spike protein receptor-binding domains from SARS-CoV-2 Beta and Kappa variants)	Aluminum hydroxide (Alum)	Standard buffers	Injection-site pain, fatigue, headache, muscle ache, fever	National approval
29	IndoVac	Indovac	PT Bio Farma, in collaboration with Baylor College of Medicine (USA) (Bandung, Indonesia)	Protein subunit, SARS−CoV−2 receptor-binding domain (RBD) spike protein	CpG 1018 (TLR−9 agonist) and aluminum hydroxide	Tris-buffered saline, polysorbate 80, sodium chloride, stabilizers	Injection-site pain, myalgia (muscle pain), fatigue, headache, and fever are less common	National approval
30	Razi Cov Pars	None	Razi Vaccine and Serum Research Institute (Karaj, Iran)	SARS−CoV−2 spike protein (includes S1, S2 subunits, and trimeric S protein)	RAS−01—oil−in−water emulsion (sesame, olive, and soybean oils + polysorbate−80)	Standard buffers	Injection-site pain, headache, mild fever	National approval
31	VidPrevtyn Beta	VAT−00002, VAT−00008	Sanofi/GSK (Paris, France)	SARS−CoV−2 spike protein (Beta variant, B.1.351 strain)	GSK’s AS03—oil−in−water emulsion containing squalene, dl−α−tocopherol, polysorbate 80	Phosphate salts, sodium chloride, polysorbate 20, water for injection; adjuvant vial: salts, water for injection; trace octylphenol ethoxylate may remain	Injection−site pain, headache, myalgia, malaise, chills; common: fever, fatigue, injection-site reactions	National approval
32	EpiVacCorona	Aurora-Cov	Vector State Research Center of Virology and Biotechnology (Koltsovo, Russia)	Chemically synthesized peptide antigens from SARS−CoV−2 S protein fragments, conjugated to recombinant N protein carrier	Aluminum hydroxide (Alum)	Formulated in phosphate-buffered saline; includes Alum; other stabilizers	Mostly pain at the injection site; occasional short fever	National approval
33	ZyCoV-D	None	Zydus Cadila (Ahmedabad, India)	Plasmid DNA encoding the SARS−CoV−2 spike protein	Unmethylated CpG motifs in the plasmid; no added external adjuvant	Phosphate-buffered saline	Injection-site pain, redness, itching, swelling, fever, muscle pain, headache, nausea, fatigue, diarrhea	National approval
34	GEMCOVAC-19	HGC019	Gennova Biopharmaceuticals Limited (Pune, India)	Self−amplifying mRNA encoding SARS−CoV−2 spike protein	None	Lipid nanoparticle formulation	Mild to moderate: injection-site pain, headache, fatigue, myalgia	National approval
35	Spikevax Bivalent Original/Omicron on BA.1	mRNA−1273.214	Moderna (Cambridge, USA)	Two mRNA components: 25 µg ela(someran) (original spike) + 25 µg imela(someran) (Omicron BA.1 spike) encapsulated in lipid nanoparticles (SM−102, PEG2000−DMG, DSPC, cholesterol)	None (self-adjuvanted mRNA in LNP formulation)	LNP lipids, sucrose, acetate buffer salts (acetic acid, sodium acetate), trometamol + trometamol HCl, sodium chloride, water for injection	Injection-site pain (~ 80%), fatigue, headache, myalgia, chills, fever, swelling/redness, nausea/vomiting, lymphadenopathy; also possible hypoaesthesia/paraesthesia, dizziness; rare myocarditis/pericarditis and capillary leak flare in predisposed individuals	National approval
36	Spikevax Bivalent Original/Omicron BA.4/BA.5	mRNA−1273.222	Moderna (Cambridge, USA)	25 µg original (elasomeran) mRNA + 25 µg Omicron BA.4/BA.5 (davesomeran) mRNA in lipid nanoparticles (SM−102, PEG2000−DMG, DSPC, cholesterol)	None (self-adjuvanted via LNP)	LNP lipids; sucrose; acetate buffer (acetic acid, sodium acetate); trometamol/trometamol HCl; sodium chloride; water for injection	Injection-site pain, fatigue, headache, myalgia, chills, fever, swelling, lymphadenopathy; rare: myocarditis/pericarditis, capillary-leak flare-ups in predisposed individuals	National approval
37	Comirnaty Bivalent Original/Omicron on BA.1	BNT162b2 bivalent (WT/Omicron BA. 4-BA.5); famtozinameran (INN)	Pfizer/BioNTech (New York, USA)	15 µg original (Wuhan-Hu-1 spike) mRNA + 15 µg Omicron BA.4/BA.5 spike mRNA, encapsulated in LNPs (ALC−0315, ALC−0159, DSPC, cholesterol, PEG−lipid)	None (self−adjuvanting via LNPs)	Lipid nanoparticles; sucrose; acetate buffer (acetic acid, sodium acetate); trometamol/trometamol HCl; sodium chloride; water for injection	Injection-site pain, fatigue, headache, myalgia, chills, fever, swelling, lymphadenopathy; rare: myocarditis/pericarditis, anaphylaxis, facial paralysis, dizziness, hypersensitivity reactions	National approval
38	Comirnaty Bivalent Original/Omicron on BA.4/BA.5	NT162b2 bivalent (WT/Omicron BA.4/BA.5); famtozinameran (INN)	Pfizer/BioNTech (New York, USA)	15 µg original Wuhan-Hu-1 spike mRNA + 15 µg Omicron BA.4/BA.5 spike mRNA encapsulated in lipid nanoparticles	None (self-adjuvanted via lipid nanoparticles)	Lipid nanoparticles (ALC-0315, ALC-0159, DSPC, cholesterol, PEG-lipid), sucrose, acetate buffer, trometamol, sodium chloride, water for injection	Injection-site pain, fatigue, headache, myalgia, chills, fever, swelling, lymphadenopathy; rare: myocarditis/pericarditis, anaphylaxis, facial paralysis, dizziness, hypersensitivity	National approval
39	AWcorna	ARCoV (previously), Walvax COVID-19 vaccine	Walvax Biotechnology and Abogen Biosciences (Kunming, China)	mRNA encoding the RBD of the SARS-CoV-2 spike protein	None (self-adjuvanted via lipid nanoparticle formulation)	Lipid nanoparticles; sucrose; acetate buffer (acetic acid, sodium acetate); trometamol/trometamol HCl; sodium chloride; water for injection	Fever, injection-site pain, fatigue, muscle pain (myalgia), headache, chills, swelling, itching (pruritus); generally mild and well-tolerated	National approval
40	iNCOVACC	BBV154	Bharat Biotech (Hyderabad, India)	Replication-deficient chimpanzee adenovirus vector (ChAd36) expressing SARS−CoV−2 spike protein (intranasal delivery)	None (vectored delivery; no separate adjuvant)	Tris buffer (pH 7.4), sodium chloride, magnesium chloride, glycerol, polysorbate 80	Headache, fever, runny nose, sneezing; rare severe allergic reactions, none in trials	National approval
41	Convidecia Air	Inhaled Convidecia, AD5−nCoV−IH	CanSino Biologics Inc. (Tiajin, China)	Replication-defective recombinant adenovirus type 5 (Ad5) vector encoding SARS−CoV−2 spike protein	None (vectored delivery; no separate adjuvant)	Formulated in saline solution for aerosolization; specific stabilizers not publicly detailed	Generally mild: fever, fatigue, headaches; fewer adverse events than intramuscular administration; no serious reactions reported in trials or real-world booster use	National approval
42	Sputnik Light	First component of Sputnik V	Gamaleya (Moscow, Russia)	Recombinant human adenovirus serotype 26 vector (rAd26) carrying SARS-CoV-2 spike gene (∼ 1 × 10^11^ viral particles)	None (viral-vector based)	Tris buffer, sodium chloride (NaCl), sucrose, MgCl_2_·6H_2_O, disodium ethylenediaminetetraacetic acid (EDTA), polysorbate 80, ethanol, water for injection	Injection-site pain, fever, headache, fatigue, muscle aches; no serious AEs reported	National approval
43	Sputnik V	Gam-COVID-Vac	Gamaleya (Moscow, Russia)	Heterologous adenoviral vector regimen: rAd26 (prime) and rAd5 (boost), both encoding SARS-CoV-2 spike protein	None (viral-vector; no separate adjuvant)	Tris buffer, sodium chloride, sucrose, magnesium chloride hexahydrate, disodium EDTA, polysorbate 80, ethanol, water for injection	Injection-site pain, fever, headache, fatigue, muscle/joint pain; rare thrombotic thrombocytopenia and isolated myocarditis/pericarditis cases reported	National approval
44	Turkovac	Erucov−Vac	Health Institutes of Turkey (Istanbul, Turkey)	β-Propiolactone-inactivated whole SARS−CoV−2 virus (traditional inactivated platform)	Aluminum hydroxide (~ 0.5 mg/dose)	Typical buffers and stabilizers (e.g., Tris, NaCl, sucrose, MgCl_2_, EDTA, polysorbate 80, water for injection)	Injection-site pain/swelling (~ 22%–27%), fatigue (~ 4%–10%), headache (~ 2%–8%), muscle/joint pain, fever (~ 1 %), rare lymphadenopathy/urticaria (~ 0.25 %)	National approval
45	FAKHRAVAC (MIVAC)	Fakhra; MIVAC	Organization of Defensive Innovation and Research (Tehran, Iran)	β-Propiolactone-inactivated whole SARS−CoV−2 virus	Aluminum hydroxide (Alum)	Standard inactivated−vaccine buffers and stabilizers (e.g., Tris, NaCl, sucrose, EDTA, polysorbate 80)	Injection-site tenderness/pain (~ 29%), headache (~ 10%), fatigue, muscle pain, nausea, vomiting, diarrhea	National approval
46	KCONVAC	Minhai COVID−19 vaccine; SARS−CoV−2 vaccine (Vero cells)	Shenzhen Kangtai Biological Products Co (Shenzhen, China)	β-Propiolactone-inactivated whole SARS−CoV−2 virus (Vero cell platform)	Aluminum hydroxide (Alum)	Standard inactivated vaccine stabilizers and buffers (e.g., PBS, sucrose, Tris, NaCl, MgCl_2_, EDTA, polysorbate 80)—typical for this platform	Injection-site pain, fatigue, headache	National approval
47	WIBP−CorV	Sinopharm WIBP	Wuhan Institute of Biological Products (Sinopharm Group, CNBG) (Wuhan, China)	β−Propiolactone−inactivated whole SARS−CoV−2 virus (strain WIV04) cultured in Vero cells; ~ 6.5 U antigen per dose	Aluminum hydroxide (~ 0.225–0.5 mg Alum per dose)	Disodium hydrogen phosphate, sodium dihydrogen phosphate, sodium chloride, phosphate-buffered saline—typical components of inactivated vaccine buffers	Injection-site pain (~ 19%–29%), fatigue, headache, low-grade fever; most symptoms resolved within 1–3 days	National approval
48	COVAX-19	Spikogen	Vaxine (Australia) in collaboration with CinnaGen (Iran) (Adelaide, Australia)	Recombinant SARS-CoV-2 spike extracellular domain protein (first recombinant spike protein trimer-based vaccine)	Advax−CpG55.2 (inulin-based polysaccharide + CpG 55.2 oligonucleotide)	Typical vaccine buffers and stabilizers (specific list not public)	Injection-site pain, headache, and fever	National approval
49	CoVLP	Covifenz	GlaxoSmithKline (GSK) (London, UK)	Virus-like particles (VLPs) of SARS-CoV-2 spike protein	AS03 adjuvant (manufactured by GlaxoSmithKline)	Potassium phosphate monobasic anhydrous, sodium chloride, sodium phosphate dibasic anhydrous, and water for injection	Chills, fatigue, joint aches, headache, mild fever, muscle aches, nasal congestion	National approval

*Alum*, aluminum-based adjuvant.

a National approval, approved only in certain countries.

## Search strategy and selection criteria

**Figure 2:**
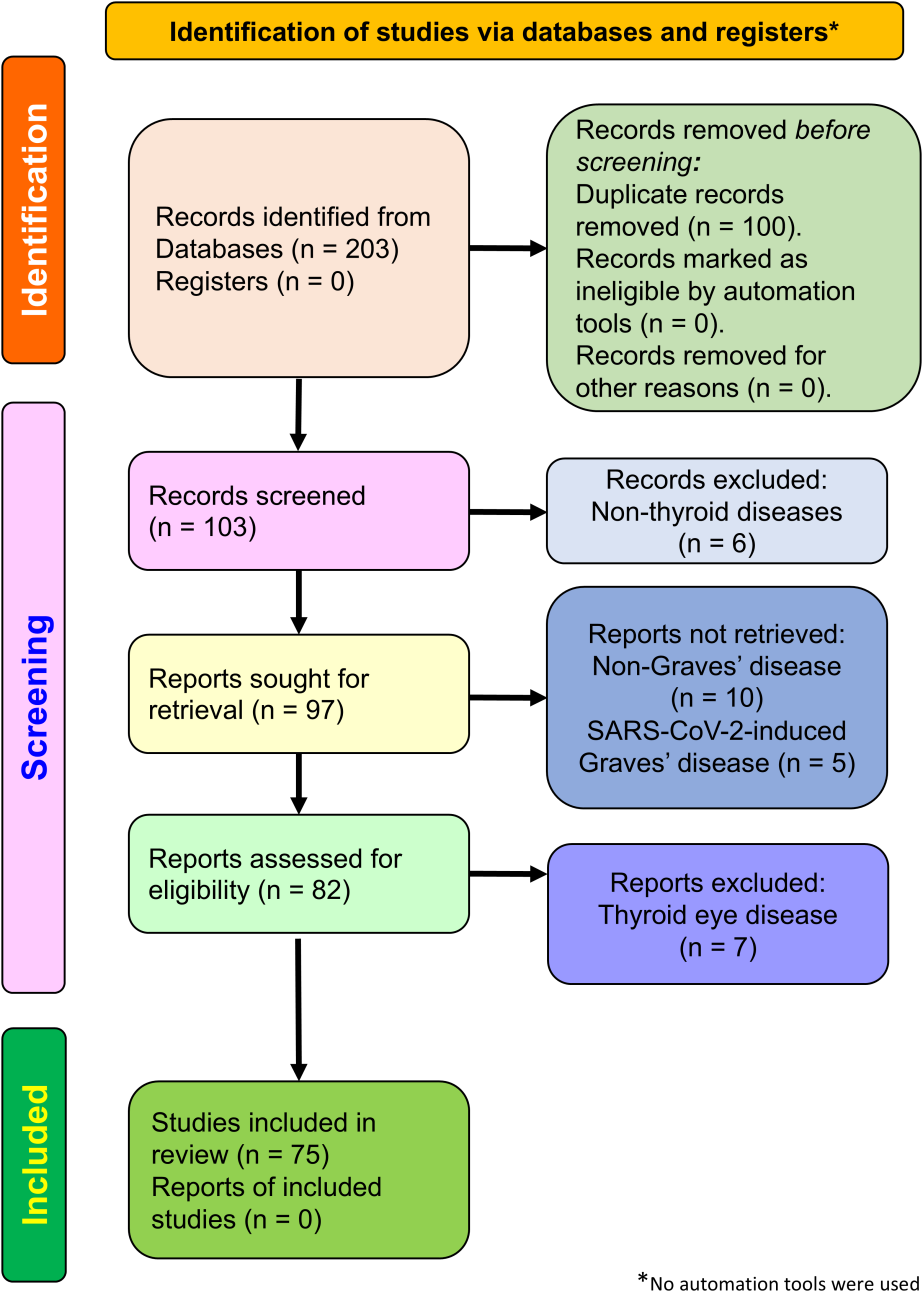
Flowchart depicting the PRISMA 2020 study selection process. The illustration shows the detailed study selection process for COVID-19 vaccine-induced hyperthyroidism of Graves’ disease.

We comprehensively searched the literature in PubMed, Web of Science, SCOPUS, and Google Scholar databases. The search terms used were “SARS-CoV-2 vaccine, Graves’ disease”, “COVID-19 vaccine, Graves’ disease”, “hyperthyroidism, COVID-19 vaccine”, and “hyperthyroidism, SARS-CoV-2 vaccine”. Only articles published in English were included. All articles published on/or before 31 December 2024 were considered, regardless of type, including full-length articles, short reports, case reports, and letters to the editor. We excluded articles reporting nonthyroid disease, non-Graves’ disease, SARS-CoV-2-induced Graves’ disease, and thyroid eye disease (TED). In total, 203 articles were retrieved through the database search. Of these, 100 duplicates, six nonthyroid disease articles, 10 non-Graves’ disease articles, five SARS-CoV-2-induced Graves’ disease articles, and seven TED articles were removed. All details are summarized in the Preferred Reporting Items for Systematic Reviews and Meta-Analyses (PRISMA) flow chart (http://www.prisma-statement.org) in **Figure 2**.

## COVID-19 vaccine induces Graves’ disease: an autoimmune hyperthyroidism

There have been many reports and studies suggesting a potential link between COVID-19 vaccination and the development of autoimmune conditions such as GD. As shown in **Table 2**, the induction period of GD in postvaccination varies depending on the vaccine type. Specifically, Pfizer-BioNTech^®^ ranges from 2 to 30 days, CoronaVac^®^ from 2 to 7 days, ChAdOx1 nCoV-19 from 5 to 46 days, and Jcovden™ from 2 to 14 days. To date, and as listed in **Table 3**, a total of 75 cases of vaccine-induced GD have been reported, and the detailed characteristics of each case are documented as described in the respective original articles (11, 21, 54–86). The characteristics of SARS-CoV-2 vaccine-induced GD are summarized in **Table 4**. The median age of affected individuals is 40 years, indicating a predilection for middle-aged adults. Out of 75 cases, 56 were women (74.7%) and 19 were men (25.3%), supporting the known female predominance in autoimmune diseases. Cases were reported globally, with the highest numbers from Singapore (15 cases), followed by Turkey (12 cases), Italy (seven cases), and the USA (six cases). These numbers may reflect the intensity of vaccination campaigns or genetic/environmental predispositions in specific populations. In total, 33 cases (44%) had a preexisting thyroid condition, such as autoimmune thyroiditis or GD in remission, whereas 42 cases (56%) had no prior thyroid-related diseases. Vaccine-associated GD occurred both with individual vaccines and through combinations of different vaccines (heterologous vaccination). The Pfizer-BioNTech^®^ BNT162b vaccine was the most frequently associated, inducing GD in 33 cases (44%), followed by ChAdOx1 nCoV-19 (Covishield, AstraZeneca) in 14 cases (18.7%), and Moderna^®^ mRNA-1273 in eight cases (10.7%). The remaining cases were linked to combinations such as Pfizer + Moderna or Sinovac with other vaccines, highlighting the effects of heterologous vaccination. Among them, 56 cases (74.7%) were new-onset, emphasizing that vaccine-induced GD is not restricted to individuals with prior thyroid disorders. This finding demonstrates the immune-activating potential of vaccines in triggering autoimmunity in some previously healthy individuals. Recurrent GD cases accounted for 18 cases (24%), representing a recurrence in those with resolved or remitted GD. Relapsed GD was identified in only one case (1.3%) involving individuals with ongoing thyroid dysfunction. Only three cases had documented concurrent thyroid diseases, such as Hashimoto’s thyroiditis, indicating that thyroid dysfunction postvaccination may primarily arise as a new autoimmune phenomenon. Onset and symptom timing were reported after the first dose in 42 cases (56%), after the second dose in 32 cases (42.7%), and after a booster dose in only one case (1.3%). This suggests a higher likelihood of onset during initial vaccine exposure, possibly due to immune priming. Major limitations of this observation include potential underreporting of cases due to mild symptoms or lack of awareness. Variations in case definitions, diagnostic criteria, and follow-up durations across studies may also influence the reported prevalence. **Table 3** highlights the predominance of new-onset GD cases, female susceptibility, and a strong association with mRNA vaccines (Pfizer-BioNTech). It also emphasizes the importance of postvaccine surveillance and patient education for timely diagnosis and management, and suggests future research directions, such as investigating genetic predispositions and mechanisms of vaccine-induced thyroid autoimmunity. **Tables 3**, **4** systematically synthesize and critically appraise the currently dispersed case reports, providing a comprehensive overview of this emerging phenomenon and highlighting potential future research directions, such as investigating genetic predispositions and mechanisms of vaccine-induced thyroid autoimmunity. **Table 5** details the hospital course and outcomes of COVID-19 vaccine-induced Graves’ disease. Symptom onset ranged from 2 to 39 days after vaccination, with common clinical features including palpitations, weight loss, heat intolerance, tremors, fatigue, and anxiety—typical manifestations of hyperthyroidism. Thyroid function tests typically revealed suppressed TSH levels with elevated free T4 and/or free T3, and over 90% of patients tested positive for TRAb, confirming the autoimmune nature of the condition. Imaging studies often showed a hypervascular thyroid on ultrasound and increased uptake on scintigraphy, consistent with active Graves’ disease. Treatment primarily involved antithyroid medications such as methimazole and beta-blockers, with corticosteroids reserved for cases with eye involvement. Most patients experienced mild to moderate illness, managed on an outpatient basis, with minimal hospitalization and no ICU admissions. The short-term outlook was favorable, with over 90% of patients demonstrating clinical and biochemical improvement within weeks to months. Long-term follow-up indicated that a minority required ongoing therapy or experienced relapse, although such instances were uncommon. Importantly, no cases of mortality or severe complications were reported. Approximately 25% of individuals had a personal or family history of thyroid autoimmunity, suggesting a potential predisposition.

**Table 2:** SARS-CoV-2 vaccines associated with the induction of Graves’ disease.

S. No.	Brand name	Generic name	Type of vaccine	Mechanism	Name of producer/company	Induction period of Graves’ disease (postvaccination)
1.	Tozinameran BNT162b2 BNT162b2 mRNA BNT162B2 Pfizer-BioNTech^®^ Pfizer^®^ COMIRNATI Tozinameran (INN)	Pfizer-BioNTech^®^ BNT162b	mRNA	A nucleoside-modified mRNA (modRNA) encoding a mutated form of the full-length spike (S) protein of SARS-CoV-2, which is encapsulated in lipid nanoparticles.	Pfizer-BioNTech	2–30 days
2.	CoronaVac^®^ CoronaVac	COVID-19 vaccine (Vero cell), inactivated	Inactivated virus	The whole SARS-CoV-2 virus, which is inactivated for vaccination	Sinovac	2–7 days
3.	AZD1222 Vaxzevria Covishield	ChAdOx1 nCoV-19	Adenoviral vector	Adenovirus (CHAdOx1) vector: this vaccine uses a chimpanzee adenovirus (ChAdOx1) as a viral vector. The adenovirus is genetically modified to be replication-deficient and carry the DNA sequence that encodes the SARS-CoV-2 spike protein.	AstraZeneca Serum Institute of India	5–39 days
4.	Moderna^®^ mRNA-1273 Elasomeran Spikevax	Moderna^®^ mRNA-1273	mRNA	Single-stranded, 5′-capped messenger RNA (mRNA) produced using a cell-free *in vitro* transcription from the corresponding DNA templates, encoding the viral spike (S) protein of SARS-CoV-2.	Moderna Inc.	5–46 days
5.	Jcovden™	Janssen COVID−19 vaccine Ad26. COV2. S	Adenoviral vector	Ad26. COV2. S: recombinant, replication-incompetent adenovirus serotype 26 (Ad26) as a viral vector. The adenovirus is modified so it cannot replicate in human cells and cause illness. The viral vector is engineered to carry the SARS-CoV-2 spike protein coding gene, which is the primary protein the virus uses to enter human cells.	Janssen Vaccines in Leiden, Netherlands	2–14 days

**Table 3:** Clinical characteristics of patients with SARS-CoV-2 vaccine-induced Graves’ disease.

Case No.	Country	Sex	Age	Symptoms	Previous history of GD/thyroid disease	Name of vaccine	Dose No.	Diagnosis after the immunization of SARS-CoV-2 vaccine	Thyroid function tests							Treatment	References	
									TSH	FT3	FT4	Anti-TSHr Ab (TRAb)	Thyroperoxidase Ab (TPOAb)	Thyrogl obulin Ab (TgAb)	Thyroid iodine uptake 24 h^a^			
1.	Mexico	F	40	Palpitation, tremor, weight loss	No	BNT162b2	1	2 days	< 0.001 mIU/L	10.5 pg/mL	3.57 ng/dL	Positive	Positive	Positive	N.A.	Conventional	Vera-Lastra et al. (11)	
2.	Mexico	F	28	Tachycardia, palpitations	No	BNT162b2	1	3 days	< 0.001 mIU/L	9.2 pg/mL	1.84 ng/dL	Positive	Positive	Positive	N.A.	Conventional	Vera-Lastra et al. (11)	
3.	USA	F	63	Pruritic rash	No	Moderna	1	7 days after first dose	0.011 µIU/mL	4.6 nmol/L	30.9 pmol/L	22 IU/L	1,149 IU/mL	N.A.	Increased activity (scintigraphy)	None	Weintraub et al. (21)	
4.	USA	M	30	Palpitation, tremor, weight loss, irritability	No	Pfizer-BioNTech	1 and 2	28 days after the second dose	< 0.005 µIU/mL	2.5 nmol/L	22.9 pmol/L	N.A.	15 IU/mL	N.A.	TSI: 0.95 IU/L	Methimazole and a beta-blocker	Weintraub et al. (21)	
5.	USA	F	71	Tachycardia, tremors	Yes, MNG	Pfizer-BioNTech	1 and 2	14 days after the second dose	< 0.01 µIU/mL	N.A.	7.2 ng/dL	N.A.	Negative	Negative	N.A., TSI, 349%	Methimazole at 20 mg twice a day and atenolol at 25 mg/day	Goblirsch et al. (54)	
6.	Italy	M	32	Anxiety, tachycardia, palpitations	No	Vaxzevria	1 and 2	10 days after the second dose	0.005 µIU/mL	7.9 pg/mL	2.96 ng/dL	7.98 IU/L	N.A.	N.A.	N.A.	Propranolol at 40 mg/day and thiamazole at 15 mg/day. Switched to propylthiouracil at 150 mg/day as he developed a rash. Improved 3 months	di Filippo et al. (55)	
7.	Italy	M	35	Headache, nausea, asthenia, palpitations, tachycardia, mild eye redness, superior palpebral retraction	No	Vaxzevria	1	5 days after first dose	< 0.004 µIU/mL	7.9 pg/mL	2.96 ng/dL	7.98 IU/L	N.A.	N.A.	N.A.	Propranolol at 20 mg/day and thiamazole at 15 mg/day	di Filippo et al. (55)	
8.	Thailand	F	33	Palpitation, weight loss	Yes	CoronaVac (2D) and ChAdOx1 nCoV-19	1 and 2	4 days	0.006 mIU/L	3.21 pg/mL	1.29 ng/dL	13.4 IU/L	N.A.	N.A.	N.A.	Methimazole at 5 mg/day	Shriphrapradang et al. (56)	
9.	USA	F	50	Eye irritation, tearing, orbital pain, proptosis, O.D.	Yes	BNT162b2 mRNA	1 and 2	3 days	Normal	Normal	N.A.	N.A.	N.A.	N.A.	N.A. (increased TSI was observed)	Teprotumumab	Rubinstein (57)	
10.	Thailand	M	70	Dyspnea, myalgia, palpitation	No	ChAdOx1 nCoV-19	1 and 2	2 days	< 0.0036 mIU/L	> 20 pg/mL	3.19 ng/dL	3.23 IU/L	N.A.	N.A.	N.A.	Methimazole at 15 mg/day	Shriphrapradang et al. (58)	
11.	Italy	M	52	Fever, weight loss, asthenia	No	BNT162B2	1 and 2	28 days	< 0.004 mIU/L	15 ng/L	5.56 ng/dL	6.48 IU/L	21 IU/mL	30 IU/mL	N.A.	Methimazole and atenolol	Patrizio et al. (59)	
12.	Japan	F	64	Fever, palpitations, respiratory distress, low urine output, edema in both legs	No	BNT162b2 mRNA	1	4 days	< 0.008 mIU/mL	23.2 ng/dL	3.3 ng/dL	33.8 IU/L	N.A.	N.A.	N.A.	Thiamazol, potassium iodine, corticosteroid, furosemide, and carvedilol. Improved in 23 days.	Yamamoto et al. (60)	
13.	South Korea	F	46	Chest pain, dyspnea	No	ChAdOx1 nCoV-19 Oxford (AstraZeneca)	1	1 day	0.010	N.A.	33.92	6.42	77.72	137.5	38.6%	N.A.	Lee et al. (61)	
14.	South Korea	F	73	Weight loss	No	ChAdOx1 nCoV-19 Oxford (AstraZeneca	2	14 days	< 0.008	N.A.	73.80	6.30	41.03	N.A.	54.2%	N.A.	Lee et al. (61)	
15.	South Korea	M	34	Weight loss, palpitation	Yes, recurrent	Janssen	1	14 days	< 0.008	N.A.	26.61	4.24	N.A	N.A	N.A.	N.A.	Lee et al. (61)	
16.	South Korea	M	39	Fever, neck pain	Yes, concurrent GD with SAT	Janssen	1	14 days	< 0.012	N.A.	36.98	2.90	< 15	295.1	13.8%	N.A.	Lee et al. (61)	
17.	Spain	F	38	Nervousness, insomnia, sweating	No	BNT162b2 mRNA	1	12 days	0.008 µIU/mL	7.46 pg/mL	2.01 ng/dL	N.A.	3,303.71 IU/mL	36.57 IU/mL	Hyperfunctioning (scintigraphy)	Methimazole	Lui et al. (62)	
18.	China	F	40	Palpitation, tachycardia	Yes	BNT162b2 mRNA	1 and 2	24 days	TSH: < 0.02 mIU/L	30.50 pmol/L	66.6 pmol/L	N.A.	239.2 kIU/L	7.2 kIU/L	Increased uptake	Carbimazole and propranolol	Lui et al. (61)	
19.	Turkey	F	40	Palpitations, sweating	No	CoronaVac and BNT162b2	1 and 2	2 days	< 0.015 mIU/L	8.79 pmol/L	27.92 pmol/L	10.3 IU/mL	195.7 IU/mL	7.1 IU/mL	Increased uptake	Methimazole at 10 mg/day	Oğuz et al. (63)	
20.	Turkey	M	29	Palpitations	No	BNT162b2	1	15 days	< 0.015 mIU/L	7.19 pmol/L	12.15 pmol/L	0.97 IU/mL	0.7 IU/mL	< 0.9 IU/mL	27% at 24 h	No	Oğuz et al. (63)	
21.	Turkey	F	43	Palpitations, sweating	Yes	CoronaVac and BNT162b2	1 and 2	9 days	0.015 mIU/L	11.4 pmol/L	33.1 pmol/L	0.25 IU/mL	0.8 IU/mL	1.8 IU/mL	61% at 24 h	Methimazole at 15 mg/day and tapered to 10 mg/day (8 weeks)	Oğuz et al. (63)	
22.	Turkey	F	43	Palpitations	Yes	BNT162b2	1	14 days	0.01 mIU/L	7.8 pmol/L	25.5 pmol/L	1.9 IU/mL	196 IU/mL	167 IU/mL	23% at 24 h	N.A.	Oğuz et al. (63)	
23.	Belgium	F	34	Swelling of eyelids, distal tremor, sweating, thermophobia, dyspnea on exertion, weight loss (after first dose)	Yes, GD remission for 7 years	BNT162b2	1 and 2	10 days after the second dose	0.01 mU/L	22.09 pmol/L	2.54 ng/dL	> 40 IU/L	N.A.	N.A.	N.A.	Thiamazole at 20 mg twice a day	Pierman et al. (64)	
24.	Turkey	F	44	Sweating, palpitation, fatigue	Yes, GD remission for 12 years	CoronaVac^®^	1	7 days	< 0.01 mIU/L	9.65 ng/L	2.67 ng/dL	12.18 IU/L	284 IU/mL	119 IU/mL	N.A.	20 mg/day methimazole (MMI) and propranolol	Bostan et al. (65)	
25.	Turkey	M	49	Palpitation, hand tremors, sweating	Yes, GD remission for 1 year	Pfizer-BioNTech^®^	1 and 2	30 days	< 0.01 mIU/L	13.50 ng/L	3.86 ng/dL	3.01 IU/L	435 IU/mL	236 IU/mL	N.A.	20 mg/day MMI and propranolol; improved in 1 month	Bostan et al. (65)	
26.	Turkey	F	31	Hot flushes, weakness, sweating	Yes, GD remission for 1 year	Pfizer-BioNTech^®^	1	21 days	< 0.01 mIU/L	21.70 ng/L	> 7.77 ng/dL	19.30 IU/L	325 IU/mL	11 IU/mL	N.A.	20 mg/day MMI and propranolol; improved in 5 weeks	Bostan et al. (65)	
27.	Turkey	F	53	Palpitations, sweating, weight loss	Yes, Hashimoto’s thyroiditis for 2 years	Pfizer-BioNTech^®^	1	7 days	< 0.01 mIU/L	8.83 ng/L	4.01 ng/dL	17.84 IU/L	55 IU/mL	1,197 IU/mL	N.A.	Propranolol and 15 mg/day MMI; improved in 2 months	Bostan et al. (65)	
28.	Turkey	F	51	Proptosis, irritation, dryness	No	Pfizer-BioNTech^®^	1 and 2	4 days	< 0.01 mIU/L	12.6 ng/L	3.72 ng/dL	5.04 IU/L	12.4 IU/mL	18.2 IU/mL	N.A.	15 mg/day MMI and propranolol, and improved in 4 months	Bostan et al. (65)	
29.	Turkey	F	47	Sweating, palpitations	No	Pfizer-BioNTech^®^	1 and 2	5 days after first dose	< 0.01 mIU/L	11.0 ng/L	3.32 ng/dL	22.74 IU/L	11.2 IU/mL	320 IU/mL	N.A.	MMI 15 mg/day and propranolol; improved in 1 month	Bostan et al. (65)	
30.	Turkey	M	46	Emotional lability, sweating, palpitation, weight loss	No	Pfizer-BioNTech^®^	1 and 2	21 days after the second dose	< 0.01 mIU/L	25.30 ng/L	> 7.77 ng/dL	9.10 IU/L	146 IU/mL	334 IU/mL	N.A.	MMI 20 mg/day and propranolol; improved in 1 month.	Bostan et al. (65)	
31.	Singapore	M	41	Tremors, palpitations	Yes, primary hyperthyroidism, but in remission	mRNA-1273 (Moderna)	1	5 days	< 0.01 mIU/L	N.A.	48.2 pmol/L	3.85 IU/L	N.A.	N.A.	N.A.	Carbimazole, restarted	Chua (66)	
32.	Singapore	F	45	Chest tightness, palpitations	No	BNT162b2 (Pfizer-BioNTech)	1	4 days	< 0.00 mIU/L5	N.A.	45.1 pmol/L	5.75 IU/L	0.3 IU/mL	N.A.	N.A.	Carbimazole	Chua (66)	
33.	Taiwan	F	39	Palpitation, hand tremor (15 days after vaccination)	No, but has an autoimmune disease	Moderna (Spikevax)	1	46 days after	< 0.0038 mIU/L	N.A.	1.54 ng/dL	42.4%	64.58 IU/mL	< 3.0 IU/mL	N.A.	Carbimazole at 10 mg once a day	Shih et al. (67)	
34.	Taiwan	F	59	Dyspnea, dizziness, palpitation (15 days after vaccination)	No, but my sister has hyperthyroidism	AstraZeneca (Vaxzevria)	1	39 days after	< 0.0038 mIU/L	N.A.	2.28 ng/dL	68.7%	< 3.0 IU/mL	1,494.7 IU/mL 8	N.A.	Carbimazole at 10 mg once a day	Shih et al. (67)	
35.	Taiwan	F	44	Tremor, heat intolerance, weight loss (4 days later)	No	AstraZeneca (Vaxzevria)	1	31 days after	< 0.0038 mIU/L	N.A.	2.74 ng/dL	80.9%	206.64 IU/mL	2,904.39 IU/mL	N.A.	Carbimazole at 10 mg once a day	Shih et al. (67)	
36.	Singapore (Chinese)	F	33	Symptoms of GD. Not specified	Yes, goiter	mRNA vaccine (Pfizer-BioNTech BNT162b/Moderna mRNA-1273)	1 and 2	7 days after first dose	0.01 mIU/L	N.A.	45 pm/L	7.3 IU/L	N.A.	N.A.	N.A.	Carbimazole at 30 mg OM, propranolol at 10 mg BD; improved in 28 days	Chee et al. (68)	
37.	Singapore (Filipino)	F	37	Symptoms of GD. Not specified	Yes, goiter (no FH of GD)	mRNA vaccine (Pfizer-BioNTech BNT162b/Moderna mRNA-1273)	1 and 2	7 days after first dose	< 0.01 mIU/L	N.A.	60 pm/L	3.8 IU/L	N.A.	N.A.	N.A.	Carbimazole at 20 mg OM, propranolol at 10 mg BD; improved in 32 days	Chee et al. (68)	
38.	Singapore (Filipino)	F	37	Symptoms of GD. Not specified	Yes, goiter (no FH of GD)	mRNA vaccine (Pfizer-BioNTech BNT162b/Moderna mRNA-1273)	1 and 2	21 days after the second dose	< 0.01 mIU/L	N.A.	72 pm/L	11.2 IU/L	N.A.	N.A.	N.A.	Carbimazole at 20 mg OM, propranolol at 10 mg BD; improved in 53 days	Chee et al. (68)	
39.	Singapore (Malay)	F	34	Symptoms of GD. Not specified	Yes, goiter (+FH of GD)	mRNA vaccine (Pfizer-BioNTech BNT162b/Moderna mRNA-1273)	1	26 days after first dose	0.01 mIU/L	23.8 pmol	68 pm/L	32 IU/L	N.A.	N.A.	N.A.	Carbimazole at 30 mg OM, propranolol at 10 mg TDS; improved in 58 days	Chee et al. (68)	
40.	Singapore (Chinese)	F	33	Symptoms of GD. Not specified	Yes, goiter (no FH of GD)	mRNA vaccine (Pfizer-BioNTech BNT162b/Moderna mRNA-1273)	1 and 2	9 days after the second dose	< 0.01 mIU/L	N.A.	29 pm/L	4.6 IU/L	N.A.	N.A.	N.A.	Carbimazole at 30 mg OM, propranolol PRN; improved in 64 days	Chee et al. (68)	
41.	Singapore (Chinese)	F	43	Symptoms of GD. Not specified	Yes, goiter (no FH of GD)	mRNA vaccine (Pfizer-BioNTech BNT162b/Moderna mRNA-1273)	1 and 2	13 days after the second dose	0.01 mIU/L	> 40 pmol	70 pm/L	6.2 IU/L	N.A.	N.A.	N.A.	Carbimazole at 20 mg OM; improved in 29 days	Chee et al. (68)	
42.	Singapore (Chinese)	M	59	Symptoms of GD. Not specified	Yes, goiter (+FH of GD)	mRNA vaccine (Pfizer-BioNTech BNT162b/Moderna mRNA-1273)	1 and 2	21 days after first dose	< 0.01 mIU/L	N.A.	49 pm/L	12.8 IU/L	N.A.	N.A.	N.A.	Carbimazole increased from 7.5 to 15 mg daily, since not improved	Chee et al. (68)	
43.	Singapore (Chinese)	F	74	Asymptomatic	Yes, goiter (+FH of GD)	mRNA vaccine (Pfizer-BioNTech BNT162b/Moderna mRNA-1273)	1 and 2	11 days after the second dose	0.02 mIU/L	N.A.	14 pm/L	6.2 IU/L	N.A.	N.A.	N.A.	Carbimazole at 2.5 mg daily	Chee et al. (68)	
44.	Singapore (Chinese)	F	25	Asymptomatic	Yes, goiter (+FH of GD	mRNA vaccine (Pfizer-BioNTech BNT162b/Moderna mRNA-1273)	1 and 2	31 days after the second dose	0.01 mIU/L	6.3 pmol	15 pm/L	2.9 IU/L	N.A.	N.A.	N.A.	Carbimazole at 2.5 mg daily	Chee et al. (68)	
45.	Singapore (Chinese)	F	41	Symptoms of GD. Not specified	Yes, goiter (no FH of GD	mRNA vaccine (Pfizer-BioNTech BNT162b/Moderna mRNA-1273)	1 and 2	21 days after the second dose	< 0.01 mIU/L	N.A.	50 pm/L	3.0 IU/L	N.A.	N.A.	N.A.	Carbimazole at 20 mg daily	Chee et al. (68)	
46.	Singapore (Chinese)	F	24	Asymptomatic	Yes, goiter (no FH of GD	mRNA vaccine (Pfizer-BioNTech BNT162b/Moderna mRNA-1273)	1 and 2	63 days after second dose	0.01 mIU/L	N.A.	20	2.4	N.A.	N.A.	N.A.	Carbimazole at 5 mg daily to 5 mg 4 times/week and 10 mg 3 times/week	Chee et al. (68)	
47.	Singapore (Chinese)	F	22	Symptoms of GD. Not specified	Yes, goiter (no FH of GD	mRNA vaccine (Pfizer-BioNTech BNT162b/Moderna mRNA-1273)	1 and 2	5 days after first dose	0.01 mIU/L	> 40	70	5.8	N.A.	N.A.	N.A.	Carbimazole at 30 mg OM, propranolol at 10 mg BD; improved in 178 days	Chee et al. (68)	
48.	Spain	F	71	Weight loss, asthenia, atrial fibrillation	No	Pfizer^®^	1 and 2	60 days after the second dose	< 0.005 mUI/L	N.A	2.3 ng/dL	3.6 U/L	30 UI/mL	< 0.9 UI/mL	Increased uptake in both lobes	Methimazole	Pla Peris et al. (69)	
49.	Spain	F	42	Weight loss, asthenia, palpitation	No	Pfizer^®^	1	10–14 days after first dose	< 0.005 mUI/L	N.A.	2.9 ng/dL	4.39 U/L	2.5 UI/mL	N.A.	Increased uptake in both lobes	Methimazole	Pla Peris et al. (69)
50.	Spain	F	54	Weight loss, asthenia, palpitation	No	Moderna^®^	1 and 2	10–14 days after second dose	< 0.005 mUI/L	N.A.	4.7 ng/dL	5.1 U/L	30 UI/mL	55 UI/mL	N.A.	Methimazole	Pla Peris et al. (69)
51.	Spain	F	46	Weight loss, palpitation, irritability	No	Pfizer^®^	1	50 days after first dose	< 0.005 mUI/L	N.A.	3.2 ng/dL	3.2 U/L	60 UI/mL	90 UI/mL	N.A.	Methimazole	Pla Peris et al. (69)
52.	Austria	F	71	Palpitations, sweating	Yes	Tozinameran	1 and 2	24 days	N.A.	11.10 pg/mL	3.56 ng/dL	4.2 IU/L	N.A.	N.A.	Mild increase	Thyreostatic	Zettinig et al. (70)
53.	Austria	F	46	Palpitations, sweating	No	Tozinameran	1	15 days	N.A.	5.18 pg/mL	1.63	2.9 IU/L	N.A.	N.A.	Normal	Thyreostatic	Zettinig et al. (70)
54.	Tunisia	F	43	Palpitation, sleep disorders, muscle weakness, heat intolerance	No	Pfizer-BioNTech	1	3 days	< 0.002	N.A.	N.A.	3.1 IU/L	N.A.	N.A.	Normal size, uniform increased uptake of isotope	Thiamazol at 30 mg and propranolol at 20 mg per day for 3 months	Taieb et al. (71)
55.	USA	M	42	Nausea, muscle weakness, shortness of breath, excessive sweating, headache, difficulty sleeping	No	Moderna	2	2 days	< 0.015	N.A.	5.96 ng/dL	16.1 IU/L	70.25 IU/mL	N.A.	Avid symmetric radionuclide uptake	Methimazole and propranolol	Singh et al. (72)
56.	USA	F	68	Nonspecific symptoms	No	Johnson & Johnson vaccine	1	2 days	0.23 mIU/mL	N.A.	1.41 ng/dL	N.A.	N.A.	N.A.	Normal uptake	Methimazole, a beta-blocker, and apixaban	Singh et al. (72)
57.	Mexico	M	57	Tremor, palpitations, weight loss, and fatigue	Goiter	Oxford-AstraZeneca SARS-CoV-2 vaccine (ChAdOx1 nCoV-19)	1	7 days	< 0.005 mIU/L	N.A.	4 ng/dL	N.A.	N.A.	N.A.	Increased diffuse, symmetric uptake	Thiamazole at 15 mg three times a day and propranolol at 20 mg twice a day	Cuenca et al. (73)
58.	Japan	F	31	Sweating, diarrhea, shortness of breath, palpitations	Yes, thyrotoxicosis at age 23; diffuse goiter	Pfizer-BioNTech (Comirnaty)	2	7 days	< 0.005	> 32.5	7.77	11.9 IU/L	481 IU/mL	82 IU/mL	Diffuse hyperaccumulation	Thiamazole (15 mg) once daily for 3 months	Sakai et al. (74)
59.	Italy	M	50	Fatigue, palpitations, distal tremor, insomnia, anxiety, nervousness, and irritability	No	Pfizer BioNTech	1	14 days	0.001 IU/mL	10.47 pg/mL	2 ng/dL	5 IU/L	529.50 IU/L	385.49 IU/L	Enlarged gland with diffuse hypercaptation of radiotracer	Methimazole (MMI, 30 mg/day)	Ruggeri et al. (75)
60.	Italy	F	55	Fatigue, tremors, and palpitations	Yes, HT	Moderna mRNA-1273	1	10 days	0.006 IU/mL	6.82 pg/mL	4.56 ng/dL	< 1.0	344 IU/L	11 IU/L	Tracer uptake was markedly reduced. compatibly with destructive thyroiditis	No treatment was prescribed	Ruggeri et al. (75)
61.	Italy	M	22	Symptoms of Graves’ disease	No	Pfizer-BioNTech	1	14 days	< 0.01	13.2	31.2	3.76 IU/L	N.A.	N.A.	Increased uptake with homogeneous distribution of the tracer	Treatment with methimazole at 20 mg/day	Manta et al. (76)
62.	India	M	20	Tremor, weight loss, and bulging of the left eye	No	ChAdox1nCoV-19 (Covishield)	1	7 days	0.002	N.A.	N.A.	2.60 IU/L	N.A	N.A	N.A.	Carbimazole at 20 mg and propranolol	Chaudhary et al. (77)
63.	India	F	46	Weight loss, heaviness in both eyes	No	ChAdox1nCoV-19 (Covishield)	1	10 days	< 0.01	N.A.	N.A.	> 40 IU/L	417 U/mL	N.A	N.A.	Carbimazole at 30 mg and propranolol	Chaudhary et al. (77)
64.	India	F	19	Hair loss, palpitation, weight loss	No	ChAdox1nCoV-19 (Covishield)	1	28 days	< 0.01	N.A.	N.A.	7.32 IU/L	703 U/mL	N.A	N.A.	Carbimazole at 10 mg and propranolol	Chaudhary et al. (77)
65.	India	F	37	Weight loss, palpitation, increased frequency of stool	No	ChAdox1nCoV-19 (Covishield)	1	14 days	< 0.01	N.A.	N.A.	4.37 IU/L	116 U/mL	N.A	N.A.	Carbimazole at 20 mg and propranolol	Chaudhary et al. (77)
66.	Greece	M	29	Palpitations	No	BNT162b mRNA vaccine	2	120 days after the second dose	0.008 mIU/L	5.07 pg/mL	1.9 ng/dL	2.9 IU/L	Negative	Negative	N.A.	Thiamazole at 5 mg/day	Lioulios et al. (78)
67.	Japan	F	41	Palpitations associated with atrial fibrillation, diarrhea, and tremor	No	Pfizer-BioNTech	2	14 days	< 0.01	33.2	72.1	5.0 IU/L	2.2 IU/ML	0.9 IU/mL	Diffuse uptake in the thyroid gland (11%)	Thiamazole and bisoprolol	Takedani et al. (79)
68.	Singapore	F	14	Palpitations, tremors, poor effort tolerance, tachycardia	No	Pfizer-BioNTech	1	14 days	< 0.01 mIU/L	> 30.80 pmol/L	70.25 pmol/L	> 40 IU/L	> 13,000 U/mL	> 2,400 U/mL	N.A.	Thiamazole at 30 mg/day with bisoprolol at 10 mg/day, administered 29 mCi of radioiodine for 9 weeks	Chen et al. (80)
69.	Italy	F	16	Symptoms of Graves’ disease	No	Pfizer-BioNTech	2	6–7 weeks								Methimazole and propranolol	Mainieri et al. (81)
70.	Taiwan	F	50	Palpitation, progressive dyspnea, dizziness, myalgia, distal tremors, and diarrhea	Yes, GD in childhood	AstraZeneca	1	10 days	0.015 IU/mL	N.A.	3.4 ng/dL	37.70%	> 1,000	N.A.	N.A.	I.V. diltiazem (15 mg), hydrocortisone (100 mg), and propranolol (30 mg). Both propranolol and carbimazole were tapered to 20 mg/day	Yan et al. (82)
71.	Taiwan	F	31	Palpitation, lump in the throat, hand tremors, dyspnea, fatigue, increased bowel movements	Yes, GD in December 2018	AstraZeneca	1	14 days	0.015 IU/mL	N.A.	4.77 ng/dL	41.29%	> 1,000	N.A.	N.A.	Carbimazole (20 mg/day) and propranolol (30 mg/day)	Yan et al. (82)
72.	Japan	M	45	Palpitations, hand tremors, and weight loss	No	Moderna	2	14 days	< 0.01 μIU/mL	27.5 pg/mL	6.42 ng/dL	17.5 IU/L	N.A.	N.A.	N.A.	Thiamazole (30 mg/day) and bisoprolol (2.5 mg/day)	Nakamura et al. (83)
73.	Japan	F	36	Palpitations	No	mRNA-1273 (Moderna)	2	28 days	< 0.01 μIU/mL	12.88 pg/mL	4.18 ng/dL	34.5 IU/L	77.0 IU/mL	38.0 IU/mL	2.21%	Thiamazole administration (10 mg/day)	Yasuda et al. (84)
74.	Turkey	F	31	Palpitations, anxiety, and weight loss	Yes, Hashimoto’s thyroiditis	2 doses of CoronaVac + 2 doses of Pfizer/BioNTech	4	30 days	< 0.003 mIU/L	N.A.	27.8 pmol/L	2.21 IU/L			Diffusely increased uptake	Methimazole and propranolol	Taşkaldıran et al. (85)
75.	Oman	F	17	Palpitation, hand tremors, and excessive sweating for a few weeks	No	Pfizer-BioNTech	1	30 days	< 0.01 m[iU]/L	> 50.00 pmol/L	> 100.00 pmol/L	N.A.	> 1,300.0 [iU]/mL	N.A.	Diffusely increased uptake. Global thyroid uptake at 27.5%	Carbimazole at 5 mg three times/day and propranolol at 40 mg three times/day	Al-Jahhafi et al. (86)

Values are given as such from the publications.

*F*, female; *FT3*, free triiodothyroinine; *FT4*, free thyroxine; *TSH*, thyroid-stimulating hormone; *Anti-TSHR Ab*, antithyroid-stimulating hormone receptor antibody; *ATD*, antithyroid drug; *Ab*, antibody.

a After administration of 100 μCi of iodine (^131^I).

**Table 4:** Characteristics of SARS-CoV-2 vaccine-induced in Graves’ disease: a summary.

Characteristics	Number of cases (%)
Total Graves’ disease cases	75 (100%)
Median age	40 years
Sex	
Male	19 (25.3%)
Female	56 (74.7%)
Country	
Austria	2 (2.7%)
Mexico	3 (4%)
Spain	5 (6.7%)
Japan	5 (6.7%)
USA	6 (8%)
Turkey	12 (16%)
Singapore	15 (20%)
Taiwan	5 (6.7%)
Italy	7 (9.3%)
Belgium	1 (1.3%)
China	1 (1.3%)
Thailand	2 (2.7%)
South Korea	4 (5.3%)
Tunisia	1 (1.3%)
India	4 (5.3%)
Greece	1 (1.3%)
Oman	1 (1.3%)
History of thyroid-related disease	
Thyroid-related disease cases	33 (44%)
No thyroid-related disease cases	42 (56%)
Type of vaccine	
Pfizer-BioNTech^®^ BNT162b	33 (44%)
COVID-19 Vaccine (Sinovac)	1 (1.3%)
ChAdOx1 nCoV-19 (Covishield, AstraZeneca)	14 (18.7%)
Moderna^®^ mRNA-1273	8 (10.7%)
Jcovden™	3 (4%)
Pfizer-BioNTech^®^ BNT162b + Moderna^®^ mRNA-1273	12 (16%)
COVID-19 Vaccine (Sinovac) + Pfizer-BioNTech^®^ BNT162b	3 (4%)
COVID-19 Vaccine (Sinovac) + ChAdOx1 nCoV-19 (Covishield, AstraZeneca)	1 (1.3%)
Disease condition	
New-onset cases	56 (74.7)
Recurrent cases	18 (24%)
Relapsed cases	1 (1.3%)
Concurrent thyroid disease	3 (4%)
Symptoms developed after	
First dose	42 (56%)
Second dose	32 (42.7%)
Booster dose (third)	1 (1.3%)

**Table 5:** Hospital course and outcomes of COVID-19 vaccine-induced Graves’ disease.

S. No.	Parameter	Findings
1.	Onset of symptoms	2 to 39 days postvaccination
2.	Common symptoms	Palpitations, weight loss, heat intolerance, tremors, fatigue, anxiety
3.	Thyroid function tests	Suppressed TSH; elevated free T4 and/or free T3
4.	Autoantibodies tested	Positive TRAb in most cases (> 90%)
5.	Imaging findings	Hypervascular thyroid on ultrasound; increased uptake on scintigraphy
6.	Treatment initiated	Antithyroid drugs (e.g., methimazole), beta-blockers, and corticosteroids if orbitopathy is present
7.	Hospitalization	Mostly mild/moderate cases; majority managed outpatient; no ICU admission (< 10%)
8.	Duration of hospital stay	Short or not required
9.	Short-term outcomes	Clinical and biochemical improvement within weeks to months (> 90%)
10.	Long-term outcomes	Some required continued therapy; rare cases of relapse (< 10%)
11.	Mortality/severe complications	None reported
12.	Relevant risk factors	Some had prior thyroid autoimmunity or family history (~ 25%)

However, it is important to note that these cases are rare, and further research is needed to establish a clear causal relationship. Autoimmune reactions may theoretically be triggered by vaccination, as the immune response stimulated by the vaccine could inadvertently target the body’s own tissues. Nonetheless, vaccines undergo rigorous testing for safety and efficacy prior to approval, and adverse effects are closely monitored. Evaluating patients’ medical histories and individual risk factors can provide critical guidance for addressing concerns regarding the COVID-19 vaccine and autoimmune conditions such as GD.

## Molecular mechanism of COVID-19 vaccine-induced Graves’ disease

Graves’ disease is an autoimmune disorder characterized by the production of TSI, which activates the TSHR and leads to hyperthyroidism. While vaccines, including those against COVID-19, play a crucial role in preventing infectious diseases; however, emerging reports suggest that, in rare cases, they may trigger autoimmune responses, including GD. The molecular mechanisms underlying COVID-19 vaccine-induced GD remain under investigation, but several hypotheses are plausible. One of the key mechanisms likely to be involved is molecular mimicry, in which vaccine components, such as the “S” protein, share structural similarities with thyroid antigens, potentially leading to cross-reactivity and the development of autoimmunity. Additionally, bystander activation can result from vaccine-induced immune stimulation, leading to the activation of autoreactive T and B cells. Adjuvant components in the vaccine formulation may further contribute to immune dysregulation, increasing the risk of autoimmunity. Molecules involved in inflammatory pathways may also play a significant role in vaccine-induced GD. Understanding these molecular pathways is essential for identifying individuals at risk and developing strategies to mitigate such adverse effects while preserving the overall benefits of COVID-19 vaccination. Further research is needed to elucidate the precise immunological triggers and improve the management of postvaccine autoimmune conditions.

## Molecular mimicry

**Figure 3:**
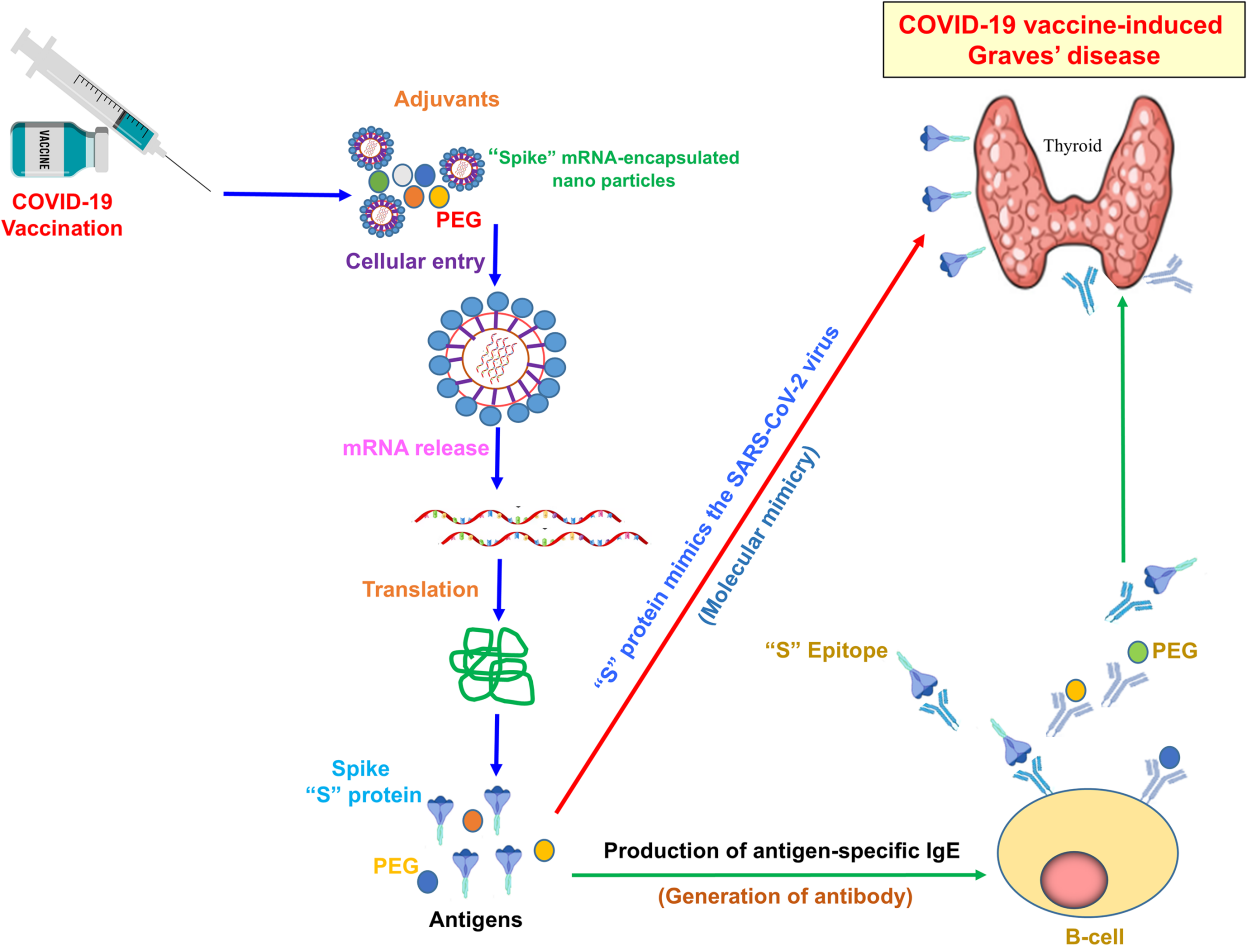
Mechanism of COVID-19 vaccine-induced Graves’ disease. The illustration typically shows how adjuvants and antigen-specific antibodies induce GD. The SARS-CoV-2 “S” protein mimics the viral antigen, triggering an immune response post-COVID-19 vaccination. Adjuvants, including PEG-encapsulated spike mRNA nanoparticles, enhance antigen delivery and immune activation. Upon mRNA release and translation, the “S” protein is expressed, leading to B-cell activation and the production of antigen-specific IgE antibodies. Molecular mimicry between the spike protein epitope and thyroid antigens may contribute to the development of autoimmune thyroid dysfunction, including GD.

Molecular mimicry, also known as the cross-reactivity theory, is a phenomenon in which antigens from infectious agents share structural similarities with self-antigens. When the immune system mounts a response against the infectious agent, it may inadvertently target self-antigens due to these similarities. As illustrated in **Figure 3**, some researchers hypothesize that molecular mimicry between antigens of the SARS-CoV-2 virus and self-antigens in the thyroid gland could contribute to the development of autoimmune thyroid disorders, including GD, following COVID-19 vaccination. In this context, the amino acid sequences of thyroid peroxidase (TPO) and the SARS-CoV-2 “S” protein share similarities (87, 88). As a result, the immune system may mistakenly produce antibodies against thyroid tissue due to its resemblance to the “S” protein generated by the mRNA vaccine. In addition, it has been observed that Japanese healthcare workers who received repeated mRNA vaccinations showed a gradual increase in mean TRAb levels (89). This rise may be attributed to cross-reactivity between thyroid tissue and the SARS-CoV-2 “S” protein, suggesting that repeated vaccinations could have induced higher TRAb production. However, the present study did not measure TPO levels in these individuals; assessing TPO alongside TRAb could provide a more comprehensive evaluation of postvaccine GD. It is important to note that, although these mechanisms are plausible, they have not been conclusively proven to contribute to COVID-19 vaccine-induced GD. On the other hand, it remains unclear whether total antibody levels are elevated in these cases. An increase in total antibodies (including anti-SARS-CoV-2 antibodies) could indicate that the rise in TRAb is part of a generalized immune activation following vaccination. In this study, no association was found between SARS-CoV-2 IgG antibodies and TRAb levels. While IgG responses are known to correlate with protection against COVID-19, assessments of cellular immunity or neutralizing antibody titers may provide a more precise connection to thyroid autoimmunity. Moreover, it is important to note that TRAb is a specific autoantibody, not typically produced during standard immune responses. A generalized increase in antibodies could theoretically unmask or trigger latent autoimmunity in genetically susceptible individuals. Therefore, while elevated total antibodies might reflect a broad immune response, the presence of TRAb indicates a more targeted autoimmune phenomenon rather than a nonspecific antibody surge. The rise in TRAb, therefore, likely reflects specific autoimmune activation. Further research is needed to elucidate the molecular mechanisms underlying this potential association.

## Antigen presentation

Vaccines, including those for COVID-19, introduce antigens—typically spike proteins or their genetic blueprints—to stimulate the immune system. After administration, antigen-presenting cells (APCs), such as dendritic cells, take up these antigens. Within the APCs, the antigens are processed into peptide fragments and loaded onto MHC class II molecules, which are then displayed on the cell surface. These MHC-antigen complexes are recognized by CD4+ T helper cells, which become activated upon binding. In most individuals, this response results in protective immunity against the virus. However, in genetically predisposed individuals, particularly those carrying autoimmune susceptibility genes such as the HLA-DRB1 haplotype, this process may inadvertently activate autoreactive T cells. These T cells, which normally remain dormant or suppressed, may then target self-antigens that resemble viral peptides due to molecular mimicry (**Figure 3**).

In Graves’ disease, the autoreactive immune response targets the TSHR, leading to the production of TRAb. These autoantibodies mimic TSH, overstimulating the thyroid and causing hyperthyroidism. Adjuvants, such as aluminum salts, used to enhance immune activation, may further exacerbate this process by promoting a stronger and longer-lasting immune response, contributing to autoimmune/inflammatory syndrome induced by adjuvants (ASIA). Graves’ disease following COVID-19 vaccination has been primarily associated with autoimmunity (62, 90, 91). Thus, COVID-19 vaccine-related GD may arise not merely from immune stimulation but from a complex interaction of antigen presentation, genetic predisposition, autoreactive T-cell activation, and heightened adjuvant-induced immune signaling. ASIA has previously been associated with autoimmune diseases following vaccinations for papillomavirus, influenza, and hepatitis B. While adjuvants are included in vaccines to enhance immune responses, they can also contribute to ASIA. A commonly used adjuvant is aluminum salt, present in vaccines such as those for papillomavirus, hepatitis B, and pneumococcal infections (89, 91, 92). In addition, genetic predisposition to ASIA has been reported, particularly among individuals carrying the HLA DRB1 haplotype (93).

## Bystander activation

In addition to specific antigen recognition, vaccines can activate immune responses through a phenomenon known as bystander activation (94). This occurs when the immune system responds to a nonspecific stimulus, such as inflammation induced by the vaccine (94). Bystander activation can trigger autoreactive T cells that may target self-antigens, including those present in the thyroid gland. T-cell antigen specificity arises from T-cell receptors (TCRs), which recognize antigen-derived peptides presented by MHC molecules on APCs (95, 96). Upon activation, T cells undergo clonal expansion and differentiate into memory cells, establishing long-term immunity. However, some T cells that are not specific to the antigen can also participate in immune responses, as they can be activated independently of antigen recognition (94, 95). This phenomenon is known as “bystander activation”. Bystander activation mainly occurs through two mechanisms. Firstly, during pathogen infections and inflammatory responses, cytokines, toll-like receptor (TLR) ligands, and other immune mediators can stimulate self-reactive T cells, potentially triggering autoimmune responses (95). Secondly, an initial inflammatory response driven by self-antigens, including activation of self-antigen-specific T cells, can lead to the bystander activation of other memory T cells with different antigen specificities. These activated T cells then release pathogenic inflammatory cytokines, contributing to the development of autoimmune diseases (96).

## Vital role of inflammatory syndrome/autoimmune factors in COVID-19 vaccine-induced Graves’ disease

This refers to the involvement of inflammatory molecules in activating the immune system during the development of GD following COVID-19 vaccination. In typical GD, the immune system targets the thyroid gland, causing overactivity and excessive production of thyroid hormones. When a person receives a COVID-19 vaccine, their immune system is stimulated to generate a response against the virus. In some individuals, however, vaccination can also trigger inflammation—partly due to vaccine adjuvants—leading to the production of autoantibodies that target the thyroid gland (75, 89). These autoantibodies can stimulate the thyroid gland to produce excessive thyroid hormones, leading to hyperthyroidism and the clinical manifestations of GD (89).

**Figure 4:**
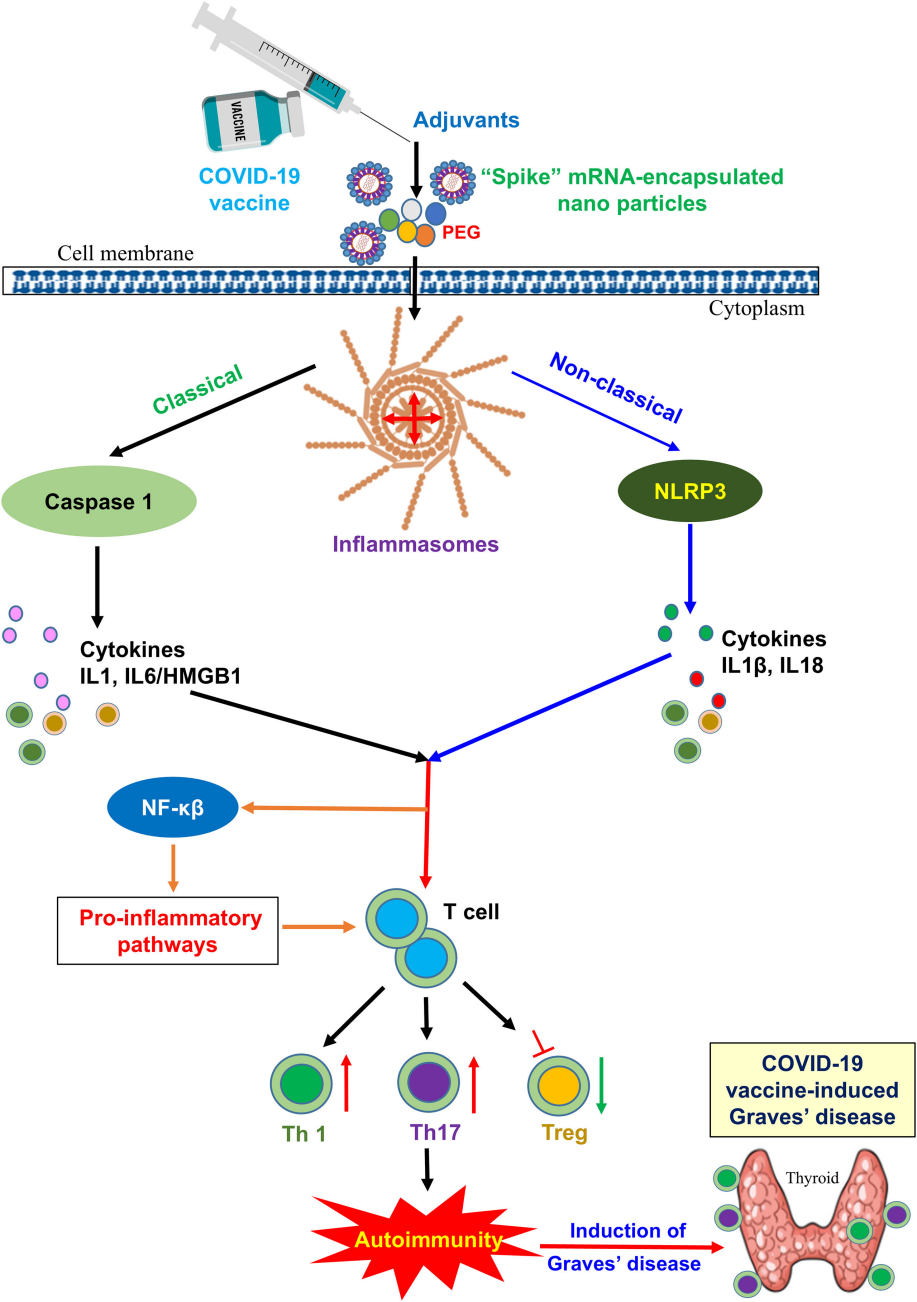
Potential mechanisms inducing autoimmunity through vaccine adjuvants: a vital role of inflammatory pathways. Vaccine adjuvants can activate inflammasomes. Canonical inflammasomes recruit caspase-1 via the adaptor molecule ASC, leading to activation of caspase-1 and the release of proinflammatory DAMPs such as IL-1α or HMGB1. Noncanonical inflammasomes activate the NLRP3 inflammasome, which indirectly induces the maturation and secretion of IL-1β and IL-18 via the noncanonical route, which binds to the receptors and activates the NF-κB signaling pathway, ultimately leading to the upregulation of NLRP3, pro-IL-1β, and pro-IL-18. In addition, preexisting antibody recognition of PEGs and direct mast cell activation, coupled with potential genetic or environmental predispositions to hypersensitivity, account for anaphylaxis to COVID-19 mRNA vaccines.

Notably, thyroid tissues have been shown to highly express ACE2, regardless of pathological condition, including cancerous stages (90). COVID-19 vaccines contain either mRNA alone (e.g., Moderna, Pfizer) or whole attenuated virus (e.g., Sinovac, Covaxin). The “S” protein produced by these vaccines binds to ACE2, leading to its downregulation, which has been found to induce the release the IL-1β and IL-6 (97). Additionally, the “S” protein may directly stimulate the production of these cytokines (98). Furthermore, mRNA vaccines contain adjuvants such as LNPs, which can trigger inflammatory cytokines, including interleukin (IL)-1β and IL-6, potentially contributing to ASIA (99, 100). The LNPs significantly increase IL-1β production by more than 22-fold and IL-6 synthesis by at least 12-fold, while generating higher antibody levels than AddaVax, an MF59-like adjuvant (98). Elevation of IL-1β and IL-6 has been shown to contribute to autoimmune disease by activating Th17 cells and suppressing regulatory T cells (88, 101) (**Figure 4**). Notably, increased IL−6 expression has been observed in patients with Graves’ disease (102). Furthermore, another study demonstrated that IL−1β induces elevated IL-6 expression in human orbital fibroblasts (103). Taken together, these findings highlight a strong mechanistic link between IL−1 and IL−6 in the pathogenesis of Graves’ disease. The development of autoimmune reactions following vaccination may be influenced by several factors, including genetic predisposition, environmental triggers, and specific characteristics of the vaccine. Understanding the role of autoimmune factors in COVID-19 vaccine-induced GD is essential for identifying individuals at risk and for developing strategies to mitigate these risks, while ensuring the continued effectiveness of COVID-19 vaccination programs.

## Benefit–risk context and rarity of post-COVID-19 vaccination Graves’ disease

It is important to acknowledge that the association between COVID-19 vaccination and the onset of Graves’ disease remains anecdotal, supported mainly by descriptive clinical reports rather than robust analytical or epidemiological data. The currently available evidence is limited to isolated case observations, which may reflect coincidental temporal relationships rather than true causality. In the absence of large-scale, controlled studies, any inference of a causal link remains speculative. Therefore, the findings summarized in this review should be interpreted with caution, considering the potential influence of publication bias and reporting variability. Our goal is not to establish causation but to consolidate existing clinical observations and propose potential immunological hypotheses that warrant further investigation. Future prospective, multicenter, and mechanistic studies are essential to confirm or refute any possible association between COVID-19 vaccination and autoimmune thyroid dysfunction.

While it is important to acknowledge the occurrence of rare autoimmune events such as Graves’ disease following COVID-19 vaccination, these cases should be interpreted within the broader context of vaccine safety and global benefit. To date, only a limited number of GD cases have been reported despite the administration of billions of vaccine doses worldwide, highlighting the exceptional rarity of this outcome. It is also plausible that some cases of vaccine-related Graves’ disease remain unrecognized, as they may be treated symptomatically without linking the onset to recent vaccination. Additionally, underreporting of mild or transient cases could lead to an underestimation of the true incidence in postvaccination surveillance data. However, the overall benefit–risk profile of COVID-19 vaccines remains highly favorable, as vaccination has played a crucial role in preventing severe disease, hospitalization, and mortality worldwide. The reporting of these rare adverse events primarily serves to enhance clinical vigilance and support timely diagnosis and management in predisposed individuals, rather than to question vaccine safety. Continued postvaccination surveillance and systematic data collection will further clarify the true incidence and immunological mechanisms underlying such uncommon autoimmune manifestations.

## Conclusions and future prospects

In conclusion, emerging reports of COVID-19 vaccine-induced GD highlight the important role of autoimmune factors in its development. The molecular mechanisms underlying vaccine-induced autoimmunity, including GD, are complex and multifactorial. Genetic predisposition, environmental triggers, and immune system dysregulation likely contribute to autoimmune reactions following vaccination. Furthermore, other recently implicated factors—such as disruptions in the gut microbiome, excessive iodine intake, stress, and epigenetic changes—further underscore the multifactorial nature of this disorder. Understanding these factors is essential for identifying individuals at risk and for developing strategies to minimize these risks while maintaining the effectiveness of COVID-19 vaccination programs. Further research is needed to elucidate the specific molecular mechanisms linking COVID-19 vaccination to the development of GD. A better understanding of these mechanisms could improve our ability to predict, prevent, and manage vaccine-induced autoimmune reactions, ultimately enhancing the safety and efficacy of vaccination efforts worldwide.
